# SIK2 kinase synthetic lethality is driven by spindle assembly defects in *FANCA*‐deficient cells

**DOI:** 10.1002/1878-0261.13027

**Published:** 2021-06-28

**Authors:** Ka‐Kui Chan, Zahi Abdul‐Sater, Aditya Sheth, Dana K. Mitchell, Richa Sharma, Donna M. Edwards, Ying He, Grzegorz Nalepa, Steven D. Rhodes, D. Wade Clapp, Elizabeth A. Sierra Potchanant

**Affiliations:** ^1^ Department of Pediatrics Riley Hospital for Children Indiana University School of Medicine Indianapolis IN USA; ^2^ Department of Medical and Molecular Genetics Indiana University School of Medicine Indianapolis IN USA; ^3^ Department of Biochemistry and Molecular Biology Indiana University School of Medicine Indianapolis IN USA; ^4^ Division of Pediatric Hematology‐Oncology Indiana University School of Medicine Indianapolis IN USA; ^5^ Present address: Department of Basic Sciences Phoenicia University Mazraat El Daoudiyeh Lebanon; ^6^ Present address: Department of Oncology St. Jude Children´s Research Hospital Memphis TN USA; ^7^ Present address: Department of Radiation Oncology University of Michigan Ann Arbor MI USA

**Keywords:** cancer, Fanconi anemia pathway, SIK2, spindle assembly checkpoint, synthetic lethality

## Abstract

The Fanconi anemia (FA) pathway safeguards genomic stability through cell cycle regulation and DNA damage repair. The canonical tumor suppressive role of FA proteins in the repair of DNA damage during interphase is well established, but their function in mitosis is incompletely understood. Here, we performed a kinome‐wide synthetic lethality screen in *FANCA* ^−/−^ fibroblasts, which revealed multiple mitotic kinases as necessary for survival of *FANCA*‐deficient cells. Among these kinases, we identified the depletion of the centrosome kinase SIK2 as synthetic lethal upon loss of *FANCA*. We found that FANCA colocalizes with SIK2 at multiple mitotic structures and regulates the activity of SIK2 at centrosomes. Furthermore, we found that loss of FANCA exacerbates cell cycle defects induced by pharmacological inhibition of SIK2, including impaired G2‐M transition, delayed mitotic progression, and cytokinesis failure. In addition, we showed that inhibition of SIK2 abrogates nocodazole‐induced prometaphase arrest, suggesting a novel role for SIK2 in the spindle assembly checkpoint. Together, these findings demonstrate that *FANCA*‐deficient cells are dependent upon SIK2 for survival, supporting a preclinical rationale for targeting of SIK2 in FA‐disrupted cancers.

## Graphical abstract

Fanconi anemia (FA) pathway disruptions are common among sporadic cancers. Thus, synthetic lethal approaches exploiting FA pathway deficiencies may present novel opportunities for the rational design of patient‐tailored strategies to improve cancer treatment outcomes. This study identifies SIK2 kinase as a synthetic lethal target of multiple FA genes, including *FANCA*. Interestingly, mitotic roles of SIK2 appear to underlie this interaction.

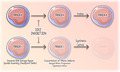

**Abbreviations**
AML: acute myeloid leukemia
DAPI: 4′,6 diamidino 2 phenylindole
FA: Fanconi anemia
FAKO: *FANCA* knockout
NHDF: normal human dermal fibroblast
SAC: spindle assembly checkpoint
TCGA: the Cancer Genome Atlas

## 1 Introduction

Fanconi anemia (FA) is a complex, heritable bone marrow failure disorder characterized by genomic instability and cancer predisposition. Canonical FA pathway signaling is essential for DNA damage repair in response to different genotoxic insults [1, 2]. Defective FA signaling impairs homologous recombination, resulting in genomic instability and increased risk of malignancy. Micronucleation and chromosome abnormalities in cells with dysfunctional FA pathway suggest that FA signaling provides an additional layer of protection from cancer by ensuring error‐free chromosome segregation during mitosis. We and others have also implicated FA signaling throughout cell cycle, including roles in centrosome function [3, 4], spindle assembly checkpoint (SAC) [4, 5], resolution of ultrafine anaphase bridges [6], and cytokinesis [7, 8].

The most commonly mutated gene in FA is *FANCA* [9, 10], one of the members forming the FA core complex, which recognizes interstrand crosslinks and induces subsequent DNA repair [11, 12, 13, 14]. Individually, FANCA is capable of catalyzing single‐strand annealing and strand exchange as an alternative repair mechanism [15]. Beyond its role in interphase, FANCA regulates mitotic centrosome function and the SAC [3, 5]. Previous studies have demonstrated that *FANCA*‐deficient cells exhibit centrosome abnormalities, chromosomal bridges, micronuclei, and multinucleation [3, 5]. Together, this evidence suggests diverse roles for FANCA throughout the cell cycle, including the regulation of mitosis.

Germline biallelic loss of FA genes is relatively rare, but germline heterozygous FA gene mutations are more common, with a subset causing haploinsufficiency, which that predisposes to cancer [16, 17, 18, 19, 20, 21]. Several studies have reported monoallelic germline mutation of *FANCA* in familial breast cancers, suggesting that *FANCA* may also be associated with breast cancer susceptibility [22, 23, 24, 25]. Furthermore, the Cancer Genome Atlas (TCGA) reports somatic disruption of the FA pathway in 30% of all cancers (Table 1) [26, 27]. Germline loss of FA pathway function renders FA patients highly susceptible to lethal toxicities from front‐line chemotherapy regimens [28]. Thus, novel synthetic lethal approaches are needed to synergize the anti‐cancer effect of standard chemotherapeutics in these patients. Additionally, the high frequency of somatic mutations within the FA pathway in cancers of the general population broadens the clinical significance of such approaches. Most notably, the use of PARP inhibitors in the treatment of *BRCA1*‐ and *BRCA2*‐deficient tumors exemplifies successful synthetic lethal targeting of the FA pathway [29, 30, 31].

**Table 1:** Somatic disruptions within the FA pathway are present in 30% of cancers documented in the TCGA PanCancer Atlas Studies. The 22 FA pathway genes were queried against 10 967 samples (10 953 patients) and were found to be altered in 3277 samples (3276 patients). Table denotes percentage of samples with alterations in each gene.

Gene symbol	Num samples altered	Percent samples altered (%)
*BRCA2*	600	5
*BRIP1*	395	4
*SLX4*	371	3
*FANCM*	366	3
*BRCA1*	357	3
*FANCA*	354	3
*FANCD2*	324	3
*FANCI*	292	2.7
*FANCB*	257	2.3
*UBE2T*	241	2.2
*PALB2*	229	2.1
*ERCC4*	229	2.1
*XRCC2*	219	2
*RAD51C*	205	1.9
*FANCG*	192	1.8
*RFWD3*	176	1.6
*FANCC*	161	1.5
*FANCE*	150	1.4
*FANCL*	145	1.3
*RAD51*	135	1.2
*MAD2L2*	116	1.1
*FANCF*	91	0.8

Through an unbiased shRNA kinome screen, we searched for gene candidates whose functions are essential for the survival of *FANCA*‐deficient cells. We identified the protein salt‐inducible kinase 2 (SIK2), an AMPK subfamily serine/threonine kinase [32], as a novel *FANCA* synthetic lethal target. SIK2 is generally regarded as a metabolic kinase that regulates glucose uptake and adipogenesis [33, 34]. However, emerging evidence implicates SIK2 in cell cycle regulation, with roles in G1/S transition, centrosome separation, mitotic progression, and cytokinesis [35, 36, 37]. Moreover, overexpression of SIK2 has been reported in different types of cancer, whereas inhibition of SIK2 was shown to suppress proliferation of ovarian and prostate cancer cells [35, 36]. Further, pharmacological inhibition of SIK2 *via* the small molecule inhibitor ARN‐3236, a derivative of 1H‐(pyrazol‐4‐yl)‐1H‐pyrrolo[2,3‐b]pyridine, has been shown to sensitize ovarian cancer cells to paclitaxel [38]. In this study, we demonstrate that ARN‐3236 causes escape from nocodazole‐induced mitotic arrest, suggesting a novel role for SIK2 in the SAC and shedding light on the underlying mechanism of sensitization to paclitaxel. We further characterize the function of SIK2 throughout mitosis and identify a functional interaction between FANCA and SIK2. Together, our findings reveal a novel FANCA‐SIK2 signaling axis that may be targeted for the treatment of FA‐associated cancers.

## 2 Materials and methods

### 2.1 Cell lines and reagents

Primary *FANCA* ^−/−^ patient fibroblasts, MNHN and JRAST, as well as their corrected counterparts, were gifts from H. Hanenberg (University of Duisburg‐Essen) and D. W. Clapp. Both isogenic cell pairs have been previously described [5]. All patient samples were obtained with the understanding and written consent of the subject and following approval by the local ethics committees (Ethikkommission der Universitaet Wuerzburg, Wuerzburg, Germany, and IRB at Rockefeller University, New York, New York, USA). The study methodologies conform to the standards set forth by the Declaration of Helsinki and were approved by the Indiana University School of Medicine Institutional Review Board (protocol #11631). Normal human dermal fibroblasts (NHDFs) and MDA‐MB231 cells were purchased from ATCC (Manassas, VA, USA). HEK293T and HeLa cells were gifts from D. Wade Clapp. THP‐1 cells were gifts from B. Gaston (Indiana University).

All cell lines except THP‐1 cells were cultured in high‐glucose Dulbecco's modified Eagle medium (DMEM; Gibco, Waltham, MA, USA) supplemented with 10% or 15% FBS (MIDSCI, Valley Park, MO, USA), 1% penicillin–streptomycin (Gibco), and 1% sodium pyruvate (Gibco). THP‐1 cells were cultured in RPMI 1640 medium (Gibco) supplemented with 10% FBS (MIDSCI), 1% penicillin–streptomycin (Gibco), and 1% sodium pyruvate (Gibco). Fibroblasts were cultured at 37 °C‐5%CO_2_‐5%O_2_ incubators to minimize oxidative damage. HeLa‐sh*FANCA* cells were generated by transducing HeLa cells with a GFP‐tagged lentivirus plasmid encoding *FANCA*‐targeting shRNA, and GFP‐positive cells were sorted out by FACS [5]. Stable *FANCA*‐knockout HeLa (HeLa‐FAKO) cells were generated by using CRISPR/Cas9 system from Santa Cruz. In brief, 1 μg *FANCA*‐CRISPR/Cas 9 plasmids and 1 μg *FANCA*‐HDR plasmid were cotransfected into HeLa cells. Stable clones were selected under 1 μg·mL^−1^ puromycin for 7 days. Stable *FANCA*‐knockdown THP‐1 cells were established by transducing with sh*FANCA* (shRNA sequence 5′CCGGCAGAGTTCTTTGTTGCTTGAACTCGAGTTCAAGCAACAAAGAACTCTGTTTTTG 3′) lentivirus plasmids (Sigma, St. Louis, MO, USA), and stable *BRCA2* knockdown MDA‐MB231 cells were generated by transducing with MISSION® shRNA Lentiviral Transduction Particles (Sigma; sh*BRCA2*‐1 shRNA sequence 5′ CCGGTACAATGTACACATGTAACACCTCGAGGTGTTACATGTGTACATTGTATTTTTG 3′; sh*BRCA2*‐2 shRNA sequence 5′ CCGGTACAATGTACACATGTAACACCTCGAGGTGTTACATGTGTACATTGTATTTTTG 3′). All transduced cells were cultured under puromycin (1 μg·mL^−1^) selection for 7 days.

Thymidine (ChemCruz) was reconstituted in PBS and filtered with 0.2 μm syringe filter (Fisher Scientific). ARN‐3236 (Greenfire Bio, Austin, TX, USA), RO3306 (Selleckchem), nocodazole (Selleckchem), and taxol (Cayman, Cayman Chemical, Ann Arbor, MI, USA) were dissolved in DMSO.

### 2.2 shRNA library screen

Primary fibroblasts derived from a *FANCA* ^−/−^ patient were used for the screen. *FANCA*‐deficient patient fibroblasts (*FANCA* ^−/−^) and their corrected counterparts (*FANCA* ^+^) were plated (two replicates/genotype) to 50% confluency in 15 cm plates (Falcon, Glendale, AZ, USA). The next day (day 1), cells were transduced per manufacturer's protocol with a lentiviral library (MISSION® LentiExpress™ Human Kinases; Sigma‐Aldrich) that encodes ∼ 5000 unique shRNA sequences targeting 513 human kinase genes (4–10 shRNA clones per gene), as well as nontargeting controls. Cells were transduced at a multiplicity of infection (MOI) of ~ 0.5. A nontransduced plate served as a negative control. On day 3, cells were selected with 1 µg·mL^−1^ puromycin media for 5 days and then allowed to grow for 2 weeks without puromycin. Cells were then pelleted and sent to Sigma for deep sequencing.

To determine the effect of each shRNA clone on cell viability, four raw copy numbers were determined for each clone representing two *FANCA* ^−/−^ replicates (1 and 2) and two *FANCA* ^+^ replicates (3 and 4). Four ratios were calculated from the raw data (1/3, 1/4, 2/3, and 2/4). Kinases with at least one shRNA showing a five‐fold copy number fold change (increase or decrease) between *FANCA* ^+^ and *FANCA* ^−/−^ cells in all four comparisons were considered significant. A ratio of < 1 was considered as synthetic lethality. Genes with at least two shRNA sequences showing a ratio < 1 with at least one of them showing five‐fold difference were chosen as potential targets for further analysis. Raw data were also processed using the Panther gene classification database to confirm significant results. Functional enrichment analysis was performed by using g:Profiler (ELIXIR) to map hits to biological processes, and pathway enrichment maps were created by Cytoscap [39]. For candidate hits, the *z*‐score of each shRNA affecting cell viability in *FANCA* deficiency was calculated by comparing the deviation of the shRNA frequency in *FANCA* ^−/−^ fibroblast from its corrected counterpart.

### 2.3 Transfection

Lipofectamine 2000 (Invitrogen, Waltham, MA, USA) was used for siRNA/shRNA and DNA transfections according to the manufacturer's instructions. For siRNA or shRNA transfection, 40–50% confluent primary patient fibroblasts or HeLa cells in six‐well format were transfected with a pool of two siRNAs at 30 nm, whereas 1 μg shRNA was used. All the siRNAs, negative control siRNA#1, siFANCA1 (s528719; 5′ ggaugguugccucuagcgutt 3′), siFANCA2 (s164; 5′ ggcacagaaauuaaaggaatt 3′), siFANCC1 (s4985; 5′ gaccagaccuuguacagautt 3′), siFANCC2 (s4986; 5′ cacucagauuugauaaagatt 3′), siBRCA1‐1 (s457; 5′ gggauaccaugcaacauaatt 3′), siBRCA1‐2 (s459; 5′ caugcaacauaaccugauatt), siBRCA2‐1 (s2085; 5′ ggauuauacauauuucgcatt 3′), siBRCA2‐2 (s224695; 5′ ggcuucaccuaaaaacguatt 3′), siSIK2‐1 (s23356; 5′ ggaagauugugcaccgugatt 3′), and siSIK2‐2 (s23357; 5′ gaaggauguugguccuagatt 3′) were purchased from Ambion (Austin, TX, USA). The siRNAs were mixed with Opti‐MEM (Gibco) and added to cells cultured with 5% serum medium for 24 h. The complete transfection process for siRNA was repeated once more before the cells were replenished with fresh, complete medium (10% serum). The cells were then harvested 48 h after the second transfection. To generate stable shRNA‐expressing cells, the viral particles were generated by transfecting HEK293T cells with the corresponding shRNA plasmids for 24 h. The viral particles were collected at 48 and 72 h after transfection. For viral transduction of THP‐1 and MDA‐MB231 cells, polybrene (8 μg·mL^−1^) was mixed with viral particles before adding them to cells. The cells were then selected 1 µg·mL^−1^ puromycin media for 5–7 days. For SIK2 overexpression, 2 μg DDK‐tagged SIK2 plasmid (RC221327; OriGene Technologies, Rockville, MD, USA) was mixed with Opti‐MEM to transfect HEK293T cells cultured with 5% serum medium for 24 h. The cells were then replenished with fresh, complete medium and incubated for 48 h before harvest.

### 2.4 MTT, CellTiter‐Glo, trypan blue exclusion, and colony formation assays

For MTT assay, 8000 cells per well were plated on a 96‐well plate. After indicated treatment, 100 µL fresh medium containing 10 µL of 5 mg·mL^−1^ MTT (Sigma‐Aldrich) was added and incubated at 37 °C for 2 h. DMSO was used to dissolve the crystals formed, and the absorbance was measured at 570 nm through a microplate reader (VersaMax, Molecular Devices, San Jose, CA, USA).

For CellTiter‐Glo cell viability assays (Promega, Madison, WI, USA), 2000 cells per well were plated in a 96‐well plate. After the indicated treatment, the CellTiter‐Glo reagent was added according to the manufacturer's instructions, and the luminescent signal was measured using Synergy H4 microplate reader (BioTek, Winooski, VT, USA).

For trypan blue exclusion assay, 10 000 cells per well were plated on a 24‐well format, and after being treated with indicated conditions, they were trypsinized and stained with 0.4% trypan blue solution (Corning, Glendale, AZ, USA). The cell number was then counted with a hemocytometer.

For colony formation assays, 500 cells per well were seeded on a six‐well plate and ARN‐3236 was added at the indicated concentrations. After 10–12 days, the cells were fixed and stained with Giemsa solution (Merck, Kenilworth, NJ, USA) containing 50% methanol. Colonies were then manually counted.

### 2.5 Immunoblotting

Cell extracts were prepared by incubating in M‐PER™ Mammalian Protein Extraction Reagent (Thermo Fisher, Waltham, MA, USA) with protease (Complete Mini, EDTA‐free; Roche, Indianapolis, IN, USA) and phosphatase inhibitors (Pierce Phosphatase Inhibitor Mini tablets; Thermo Scientific) on ice. Lysates were then centrifuged at top speed in a microcentrifuge for 10 min to remove cellular debris. Protein concentrations were measured using Pierce™ BCA protein assay kit (Thermo Fisher), and samples were diluted with LDS sample buffer (NuPAGE; Life technologies, Carlsbad, CA, USA). Proteins were separated by electrophoresis on 4–12% Bis‐Tris gels (Invitrogen) and transferred to PVDF membranes (Millipore, Burlington, MA, USA). After blocking in 5% nonfat milk, the membranes were probed with primary antibodies at 4 °C overnight and then the corresponding secondary antibodies (IRDye, LI‐COR) for another hour at room temperature before the images were captured by Odyssey CLx imager (LI‐COR). image studio 2.1 software was used to quantify protein levels by measuring the relative fluorescence intensities of bands (normalized against loading control). The primary antibodies used included rabbit anti‐Akt (Cell Signaling Technology, Danvers, MA, USA), rabbit anti‐phospho‐Akt (Cell Signaling Technology, Danvers, MA, USA), rabbit anti‐Aurora B (Abcam, Cambridge, UK), mouse anti‐β actin (Sigma), rabbit anti‐GAPDH (Cell Signaling Technology), rabbit anti‐Cyclin B1 (Cell Signaling Technology), rabbit anti‐DDK (Cell Signaling Technology), rabbit anti‐FANCA (Abcam), rabbit anti‐SIK2 (Cell Signaling Technology), and rabbit anti‐pS358 SIK2 (Kinexus, Vancouver, Canada).

### 2.6 Cell cycle analysis and phospho‐Histone H3 (Ser10) staining

After indicated conditions with ARN‐3236, cells were pelleted and fixed with 70% ethanol at 4 °C overnight. For cell cycle analysis, FxCycle PI/RNase staining solution (Invitrogen) was used to stain the cells at 4 °C overnight. For phospho‐H3 staining, cell pellets were washed with PBS once and resuspended in PBS containing 0.25% Triton X‐100 on ice for 15 min. The pellets were then stained with rabbit anti‐phospho‐Histone H3 antibody (1 : 1000; Abcam) in 1% BSA at 4 °C overnight. After washing with PBS, the cell pellets were probed with anti‐rabbit Alexa Fluor 488 secondary antibody (Invitrogen) for 1 h. The pellets were counterstained with propidium iodide (25 μg·mL^−1^) before analysis by flow cytometry (FACSCanto II; BD Biosciences, Franklin Lakes, NJ, USA).

### 2.7 Live‐cell imaging

Twenty thousand cells per quadrant were plated in a 35mm Hi‐Q4 culture dish (Ibidi, Planegg, Germany). ARN‐3236 was added, and time‐lapse phase‐contrast images (4 z‐sections, 2 μm each) were acquired every 2 min for a total of 24 h using a BioStation IM‐Q time‐lapse imaging system (Nikon, Tokyo, Japan) fitted with a temperature‐controlled growth chamber. Video analysis was done through nis‐elements viewer 4.50 imaging software (Nikon), and Imaris (Bitplane, Zürich, Switzerland) was used to export the video.

### 2.8 Co‐immunoprecipitation

Immunoprecipitation was performed using Pierce Crosslink Immunoprecipitation Kit (Thermo Scientific) according to the manufacturer's instruction with modifications. In brief, approximately 1 × 10^7^ cells were lysed using lysis/wash buffer, and the lysates were precleared with control agarose resin for an hour at 4 °C. Cell lysates were collected after centrifugation and added to Protein A/G Plus Agarose resin premixed with antibody in coupling buffer. The mixtures were incubated with agitation at 4 °C overnight. The resin was then washed sequentially with lysis/wash buffer and TBS. The bound antigens were eluted by mixing the resin with sample buffer and incubated at 95 °C for 5 min. The eluate was collected after centrifugation at 7200 ***g*** for 2 min.

### 2.9 Immunofluorescence

Cells were grown on glass coverslips (Fisher Scientific) under indicated conditions. Cells were pre‐extracted with 0.2% Triton X‐100/PBS for 15 s and then fixed with 4% paraformaldehyde/PBS for 10 min. For mitotic spindle detection, cells were pre‐extracted and fixed with PHEM buffer containing 0.2% Triton X‐100 and 4% paraformaldehyde. After fixation, cells were washed with PBS, permeabilized with 0.5% Triton X‐100 in 5% BSA/PBS for an hour, and then incubated in primary antibodies (1 : 100) in 5% BSA/PBS at 4 °C overnight. Cells were then washed with 1% BSA/PBS three times and probed with fluorophore‐conjugated secondary antibodies (1 : 2000 in PBS) for 2 h at room temperature. The coverslips were mounted with ProLong Diamond™ antifade mountant with 4′,6‐diamidino‐2‐phenylindole (DAPI; Invitrogen) on slides (Fisher Scientific). Primary antibodies used include mouse anti‐SIK2 (BioLegend, San Diego, CA, USA), mouse anti‐SIK2 (Abcam), rabbit anti‐SIK2 (Cell Signaling), rabbit anti‐SIK2‐pS358 (Kinexus), monoclonal rabbit anti‐FANCA (Abcam), polyclonal rabbit anti‐FANCA (Abcam), goat anti‐FANCA (R&D Systems, Minneapolis, MN, USA), goat anti‐Pericentrin 2 (Santa Cruz), rat anti‐α Tubulin (Abcam), mouse anti‐C‐Nap1 (Millipore), and human autoantibody against centromere (CREST; ImmunoVision, Springdale, AR, USA).

The fluorescence signals were captured by a Deltavision Ultra microscope using 60× or 100× lenses (GE Healthcare, Chicago, IL, USA). Images were acquired with z‐section of 0.2 μm each and deconvolved using Softworx (GE healthcare). All images were processed with Imaris (Bitplane). Images in figures represent individual z‐sections of deconvolved stacks. The ‘interpolate zoom’ function in Imaris was used to smooth images.

### 2.10 Double thymidine block and RO3306 induced G2 arrest

Cells were treated with 2.5 mm thymidine solution for 24 h. The cells were then released for 8 h and blocked again with 2.5 mm thymidine for another 16 h. Cells were released, and cell lysates were collected at different time points as indicated. For RO3306 treatment, 9 µm RO3306 was added to cells grown on coverslips for 20 h. After extensive washing, the cells were released and ARN‐3236 was added for 4 h. The cells were fixed and processed as mentioned in fluorescence microscopy. The coverslips were stained with Alexa Fluor 594 phalloidin (Life technologies) for 2 h and then mounted.

### 2.11 Statistical analysis

graphpad prism8 (San Diego, CA, USA) was used to perform the statistical analyses, and *P* value < 0.05 was considered as significance. The exact *P* values with significance are denoted on the graph.

## 3 Results

### 3.1 Identification of kinases crucial for survival of *FANCA*‐deficient cells

**Fig. 1:**
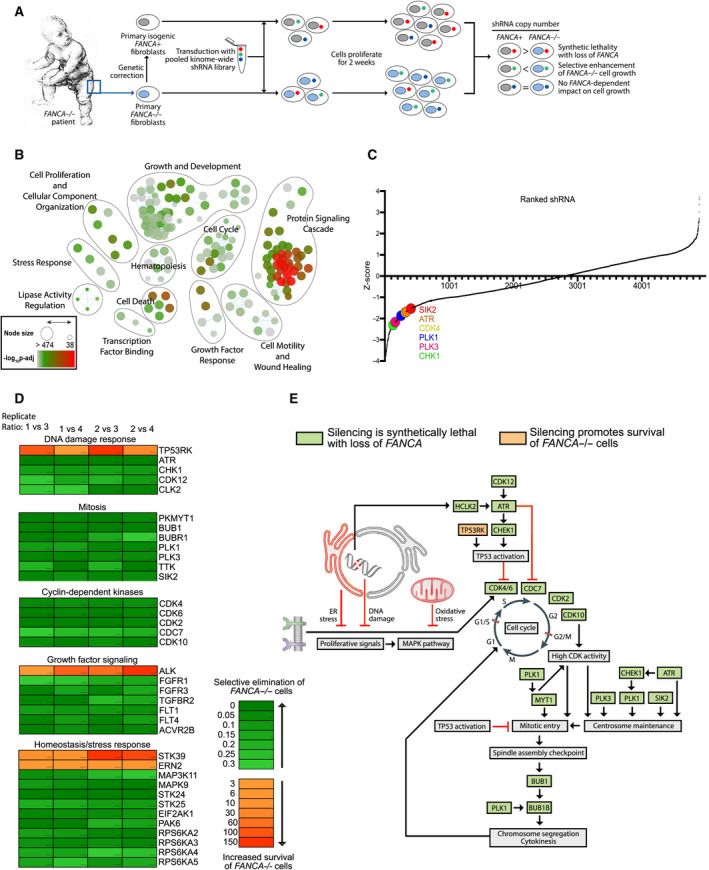
Screen and pathways analysis. (A) Workflow of kinome screen targeting > 500 human kinases conducted in *FANCA* ^−/−^ patient fibroblasts and their gene correction counterpart. Patient fibroblasts (*FANCA* ^−/−^ *)* and the corrected fibroblasts (*FANCA* ^+^ *)* were transduced with the lentivirus‐based shRNA pool (> 5000 clones), cultured in puromycin for 5 days to select for transduced cells, and then allowed to grow for 2 weeks in puromycin‐free media. The cells were collected, and the copy number of individual clones was analyzed by high‐throughput sequencing. Two biological replicates for each *FANCA* genotype were used. (B) Node plot categorizing gene hits into significantly enriched biological processes (*P* < 0.01). The detailed gene list of each biological process is shown in Data Set S1. (C) Scatter Plot of *z*‐score for each shRNA in the screen. Select synthetic lethal hits of interest (e.g., PLK1, SIK2, ATR, and Chk1) are indicated as enlarged, colored dots. The *z*‐score for SIK2 is −1.53. (D) Representative candidate genes were grouped and categorized according to their functional roles in mitosis and several other biological processes. The values represented by the four columns in the heat map were generated by calculating the ratio of the copy number of each shRNA in one *FANCA* ^−/−^ replicate (samples #1 and #2) to that of the shRNA copy number in one *FANCA* ^+^ replicate (samples #3 and #4). Thus, the four columns represent the four ratios generated (i.e., sample#1/sample#3, sample#1/sample#4, sample#2/sample#3, sample#2/sample#4). (E) A diagram demonstrating the link between cell cycle and other cellular pathways as well as how screen‐identified candidate genes perform their cell cycle‐related functions.

Primary patient‐derived *FANCA* ^−/−^ fibroblasts and their gene‐corrected counterparts (*FANCA* ^+^) were used to carry out a lentivirus‐based shRNA kinome screen (Fig. 1A). *FANCA* ^−/−^ and *FANCA* ^+^ cells were transduced with a kinome‐wide lentiviral library containing approximately 5000 distinct shRNAs targeting over 500 human kinases. Cells were transduced with an MOI of 0.5 so that each cell should receive no more than one shRNA clone. Kinases with at least one shRNA with a 5‐fold copy number change between *FANCA* ^+^ and *FANCA* ^−/−^ cells were deemed significant. Using this criterion, we identified 87 genes as essential to *FANCA*‐deficient cell survival (i.e., the copy number of at least one shRNA targeting these kinases was five‐fold less in *FANCA* ^−/−^ cells relative to *FANCA* ^+^ cells). Functional enrichment analysis was performed by using g:Profiler (ELIXIR) to map hits to biological processes, and pathway enrichment maps were created by Cytoscape (Fig. 1B)[39]. The identified gene list and their corresponding pathway enrichment analysis are shown in Data Set S1. Among these hits were multiple DNA damage response (DDR) kinases previously identified by others as FA synthetic lethal targets, including *ATR* [41], *CHK1* [42, 43], and *CDK12* [44] (Fig. 1D,E). These independent findings consistently demonstrate the functional significance of FA genes in the DDR pathway and serve as internal validation of our experimental approach (Fig. 1A). In addition, ontology analysis showed enrichment of myriad biological process clusters, including cell cycle regulation, implying an increased reliance on mitotic governance in *FANCA* ^−/−^ cells (Fig. 1B,E). *Z*‐score analysis of shRNA copy number revealed that most genes identified as synthetic lethal by five‐fold exclusion clustered together with *z*‐scores < −1.5 (Fig. 1C; Data set S1). Hits with known mitotic functions included *SIK2*, which, like *FANCA*, has been specifically implicated in centrosome maintenance and cytokinesis [5, 8, 35, 36]. Because of this functional overlap, we reasoned that further characterization of the synthetic lethal interaction between *FANCA* and *SIK2* may provide mechanistic insight into the mitotic function of FANCA.

**Fig. 2:**
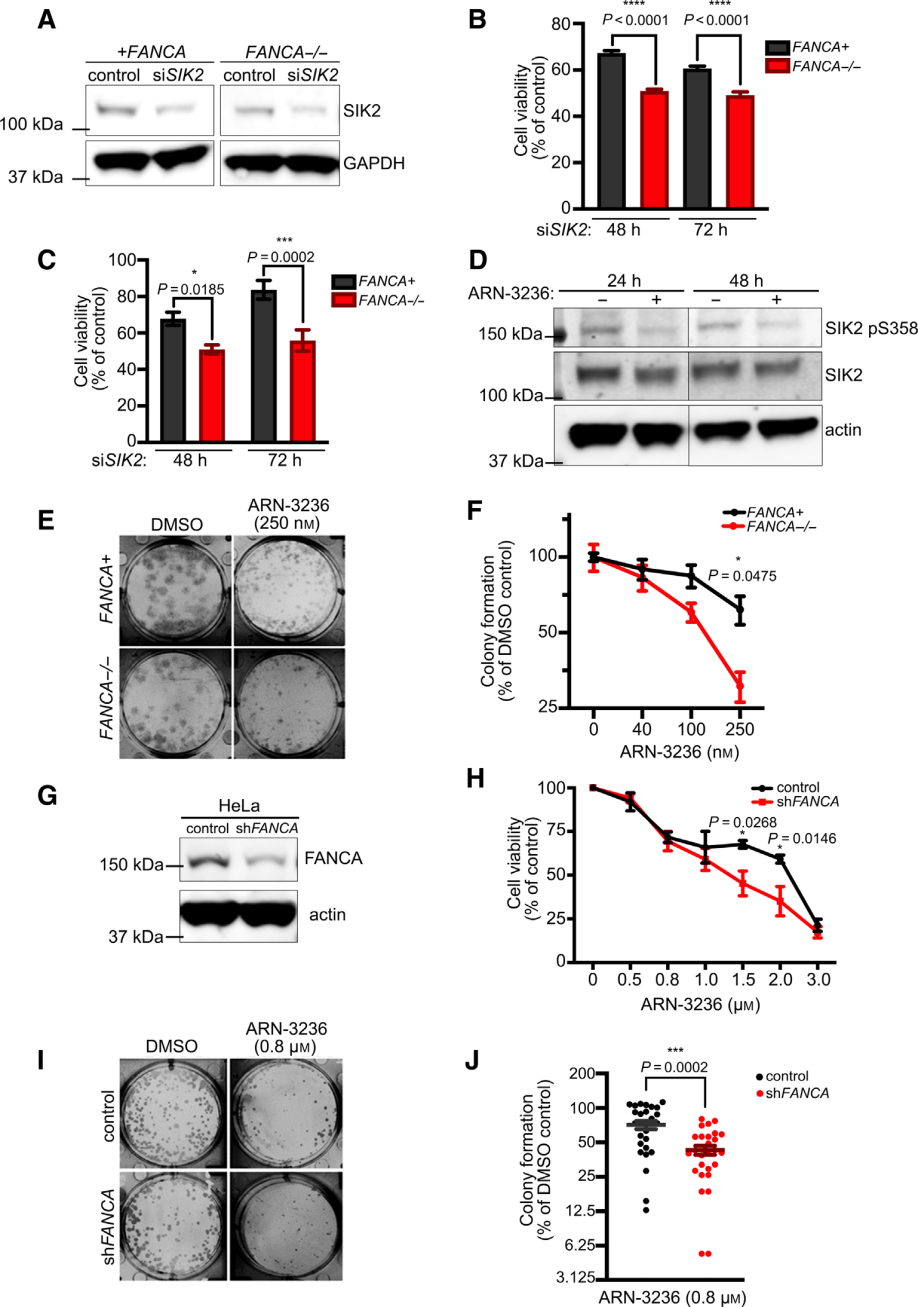
*SIK2* is a synthetic lethal target of *FANCA*. (A) Representative western blot image showing knockdown of *SIK2* in *FANCA* ^−/−^ patient fibroblasts and *FANCA* ^+^ counterpart. Relative to nontargeting control siRNA, *SIK2*‐targeting siRNA reduced SIK2 expression by an average of 53% and 48% in *FANCA* ^−/−^ and *FANCA* ^+^ fibroblasts, respectively. Data are representative of two independent experiments. (B) Quantification of patient fibroblast viability after *SIK2* knockdown as assessed by MTT assay. Plot shows mean ± SEM of data pooled from three independent experiments. *P* values were calculated by one‐way ANOVA. (C) Quantification of patient fibroblast viability after *SIK2* knockdown as assessed by trypan blue exclusion assay. Plot shows mean ± SEM of data pooled from three independent experiments. *P* values were calculated by two‐way ANOVA. (D) Representative western blot showing reduced levels of SIK2 pS358 in *FANCA* ^+^ patient fibroblasts after treatment with 2 μm ARN‐3236. Expression of the SIK2 pS358 in *FANCA* ^+^ fibroblasts is decreased by an average of 65% and 60% after 24‐ and 48‐h treatment with ARN‐3236 relative to DMSO control. A representative blot of two independent experiments is shown. (E) Representative colony formation assay in patient fibroblasts treated with ARN‐3236. (F) Quantification of fold change in colony formation of ARN‐3236‐treated patient fibroblasts relative to DMSO control. Graph shows mean ± SEM of data pooled from three independent experiments, plotted in Log_2_ scale. *P* value was calculated by two‐way ANOVA. For *FANCA* ^+^ vs *FANCA* ^−/−^ treated with 250 nm ARN‐3236, *P* = 0.0475. (G) Representative western blot confirming shRNA‐mediated knockdown of *FANCA* in HeLa cells. HeLa‐sh*FANCA* showing 65% decrease in FANCA expression when compared to control HeLa cells. A representative blot of two independent experiments is shown. (H) Dose‐dependent response of sh*FANCA* HeLa cells treated with ARN‐3236. The cell viability of sh*FANCA* HeLa cells treated with ARN‐3236 for 48 h was measured by MTT assay. Plot shows mean ± SEM of data pooled from three independent experiments. *P* values were calculated by two‐way ANOVA. *P* values for control vs sh*FANCA* HeLa treated with 1.5 and 2 μm ARN‐3236 equal 0.0268 and 0.0146, respectively. (I) Representative colony formation assay in control and stable *shFANCA* HeLa cells treated with ARN‐3236. (J) Quantification of fold change in colony formation of ARN‐3236‐treated control and stable *shFANCA* HeLa cells relative to DMSO control. Graph shows mean ± SEM of data pooled from five independent experiments, plotted in Log_2_ scale. *P* = 0.0002, calculated by unpaired *t*‐test.

**Fig. 3:**
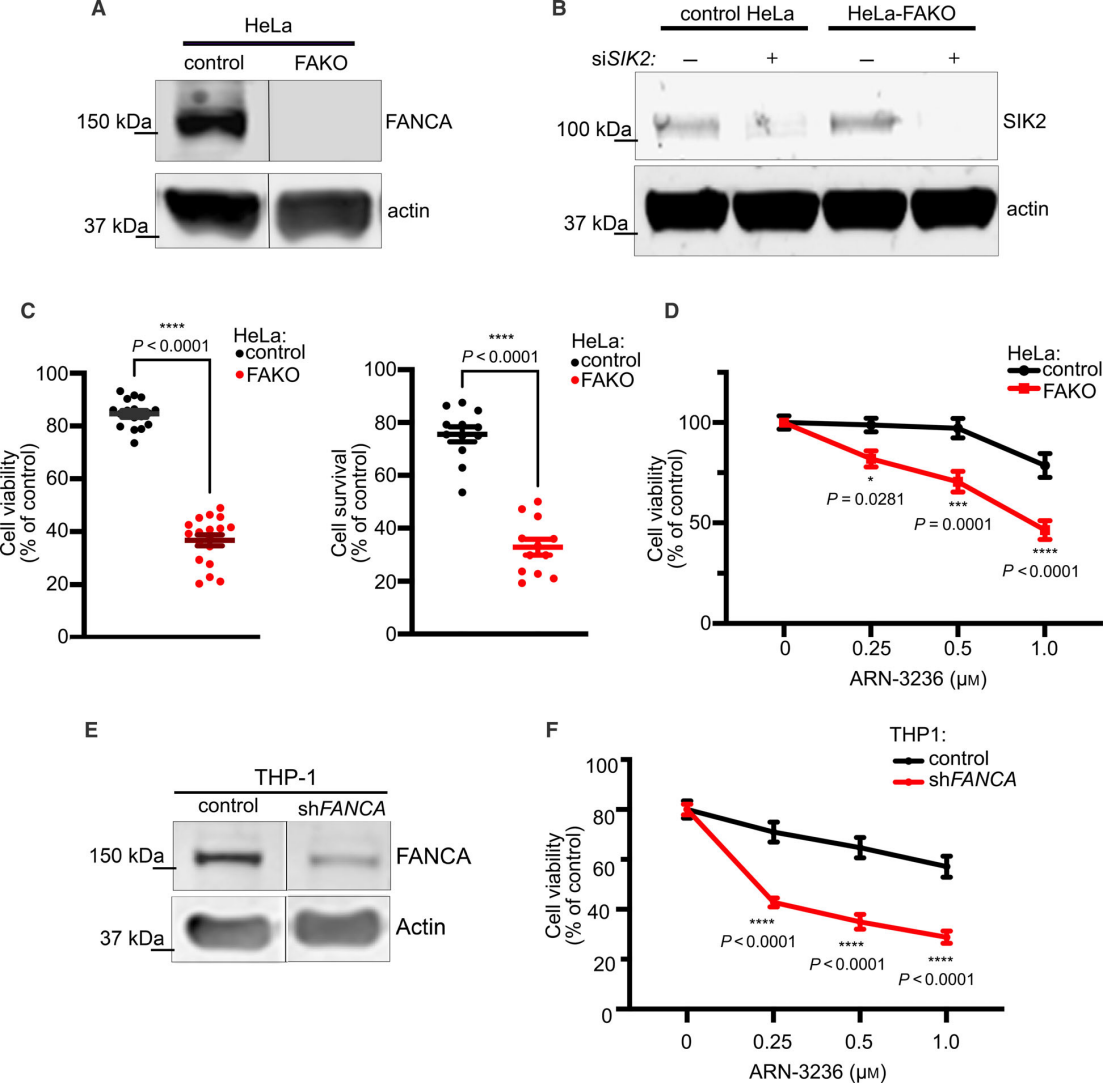
Depletion or inhibition of SIK2 is synthetic lethal in FAKO HeLa cells. (A) Representative western blot showing reduced SIK2 expression in *FANCA*‐CRISPR HeLa cells (HeLa‐FAKO) and control HeLa cells. (B) Representative western blot of HeLa‐FAKO cells transfected with si*SIK2*. An average 49% and 42% decrease of SIK2 were seen in control HeLa and HeLa‐FAKO cells transfected with si*SIK2*, respectively, from three independent experiments. (C) Response of FAKO HeLa‐FAKO cells transfected with si*SIK2*. The cell viability was measured by and MTT (left) trypan blue exclusion (right) assays, respectively. Representative plots show mean ± SEM of data from three independent experiments. *P* < 0.0001, calculated by one‐way ANOVA. (D) Response of HeLa‐FAKO treated with ARN‐3236 for 48 h. The cell viability was measured by MTT assay. Plots show mean ± SEM of data pooled from three independent experiments. For control vs FAKO HeLa cells treated with 0.25, 0.5, and 1 μm ARN‐3236, *P* values equal 0.0281, 0.0001, and < 0.0001, respectively, as calculated by two‐way ANOVA with ad hoc Sidak's multiple comparison test. (E) Representative western blot showing reduced FANCA level in THP‐1 cells after transducing with sh*FANCA*. An average 61% decrease of FANCA from two independent experiments was seen in THP‐1‐sh*FANCA* cells. (F) Dose‐dependent response of sh*FANCA* THP‐1 cells treated with ARN‐3236. The cell viability of THP‐1‐sh*FANCA* cells treated with ARN‐3236 for 72 h was measured by CellTiter‐Glo assay. Representative plot shows mean ± SEM of data from four independent experiments. For control vs sh*FANCA* THP‐1 cells treated with 0.25, 0.5, and 1 μm ARN‐3236, all *P* values < 0.0001, as calculated by two‐way ANOVA with ad hoc Sidak's multiple comparison test. *P* values were calculated by two‐way ANOVA with ad hoc Sidak's multiple comparison test.

To validate that *FANCA* ^−/−^ cells are dependent upon *SIK2* for survival, a pool of two screen‐independent *SIK2*‐targeting siRNAs was used to knock down *SIK2* in *FANCA* ^−/−^ patient fibroblasts, as well as their gene‐corrected counterparts (Fig. 2A). *FANCA* ^−/−^ cells exhibited significantly decreased viability following *SIK2* knockdown relative to *FANCA* ^+^ cells, as measured by MTT and trypan blue exclusion assays (Fig. 2B,C). To determine the effect of pharmacological inhibition of SIK2 on *FANCA* ^−/−^ cell survival, we employed the SIK2‐specific small molecule inhibitor ARN‐3236. Following ARN‐3236 treatment, SIK2 inhibition was confirmed by western blot analysis of SIK2 serine‐358 phosphorylation (SIK2 pS358), a marker of SIK2 activity [38, 45]. Indeed, ARN‐3236‐treated patient fibroblasts and HeLa cells exhibited reduced levels of SIK2 pS358 relative to DMSO‐treated controls (Fig. 2D and Fig. S1A). In addition, phosphorylation of AKT, a SIK2 downstream target, was also reduced in HeLa cells treated with ARN‐3236, corroborating its inhibition of SIK2 kinase activity (Fig. S1B). ARN‐3236 treatment significantly reduced colony formation of *FANCA* ^−/−^ cells relative to *FANCA* ^+^ cells (Fig. 2E,F). To validate this result in an independent cell type, we assessed colony formation of HeLa cells stably expressing *FANCA*‐targeting shRNA or nontargeting shRNA following treatment with ARN‐3236 (Fig. 2G). Similarly, ARN‐3236 more severely decreased cell viability and colony formation in *FANCA*‐knockdown cells relative to control cells (Fig. 2H–J). We also established stable FAKO HeLa cells using CRISPR gene editing (Fig. 3A) and demonstrated that concomitant knockdown of *SIK2* significantly decreased viability in HeLa‐FAKO compared to HeLa CRISPR control (Fig. 3B,C). Consistently, HeLa‐FAKO cells were hypersensitive to ARN‐3236 (Fig. 3D). Since FA patients display a high risk of childhood acute myeloid leukemia (AML), we stably knocked down *FANCA* in the AML cell line THP‐1 to assess sensitivity to SIK2 inhibition in a relevant cancer type. Importantly, knockdown of *FANCA* significantly sensitized THP‐1 cells to ARN‐3236 (Fig. 3E,F). Together, these findings demonstrate that *FANCA*‐deficient cells are specifically sensitive to depletion or inhibition of SIK2.

### 3.2 Loss of FANCA exacerbates cell cycle defects induced by SIK2 inhibition

**Fig. 4:**
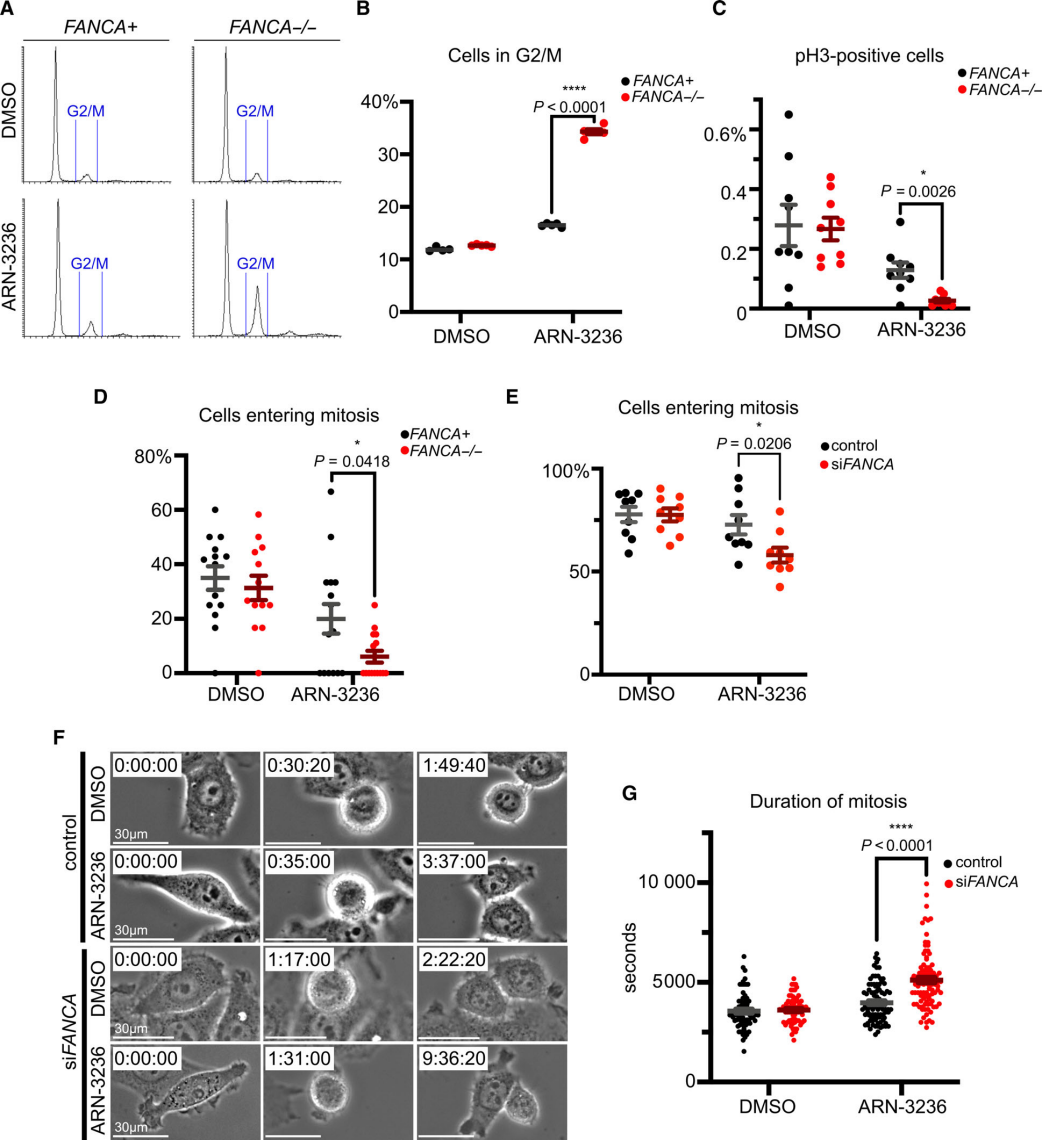
Cell cycle disruption induced by SIK2 inhibition is enhanced in FANCA‐deficient cells. (A) Representative histograms showing cell cycle profiles of patient fibroblasts following 3‐day treatment with ARN‐3236 (3 μm). (B) SIK2 Inhibition induced greater G2/M phase arrest in *FANCA* ^−/−^ cells than *FANCA* ^+^ cells. Quantification shows significantly greater G2/M population in *FANCA* ^−/−^ cells after ARN‐3236 treatment. Plot shows mean ± SEM of data pooled from two independent experiments. For each experiment, 10 000 cells per condition were analyzed. For *FANCA* ^+^ vs *FANCA* ^−/−^ cells treated with ARN‐2326, *P* < 0.0001, as calculated *via* one‐way ANOVA. (C) Quantification of percent cells positive for phospho‐H3 staining after treatment with ARN‐3236 (3 μm) for 3 days. Data are pooled from three independent experiments. For each experiment, 10 000 cells per condition were analyzed. *P* value was calculated *via* multiple *t*‐tests and adjusted for multiple comparisons using the Holm–Sidak method. For *FANCA* ^+^ vs *FANCA* ^−/−^ cells treated with ARN‐2326, *P* = 0.0206. (D) Quantification of the percentage of patient fibroblasts that entered mitosis over a 24‐h period in the presence of ARN‐3236 or DMSO. Patient fibroblasts were treated with ARN‐3236 (2 μm) and monitored for 24 h. Plot shows mean ± SEM of data pooled from three independent experiments. At least 16 cells per condition were analyzed for each experiment. For *FANCA* ^+^ vs *FANCA* ^−/−^ cells treated with ARN‐2326, *P* = 0.0418, as calculated by two‐way ANOVA. (E) Quantification of percentage of HeLa cells with or without *FANCA* silencing that entered mitosis during a 24‐h period in the presence of ARN‐3236 (1 μm) or DMSO. Plot shows mean ± SEM of data pooled from three independent experiments. At least 63 cells per condition were analyzed for each experiment. *P* value was calculated by two‐way ANOVA. For control vs si*FANCA* HeLa cells treated with ARN‐3236, *P* = 0.0206. (F) Representative stills from live imaging of *FANCA*‐knockdown vs control HeLa cells treated with ARN‐3236 (1 μm) or DMSO. Time stamps marking initiation of cell rounding (first column) and flattening (third column) indicate duration of mitosis. Scale bars represent 30 μm. (G) Quantification of mitotic duration in *FANCA*‐knockdown vs control HeLa cells in the presence of ARN‐3236 (1 μm) or DMSO. Plot shows the mean ± SEM of data pooled from three independent experiments. At least 72 cells per condition were analyzed. *P* value was calculated by one‐way ANOVA. For control vs si*FANCA* HeLa cells treated with ARN‐3236, *P* < 0.0001.

*SIK2* depletion has been shown to arrest cells in G2 and block mitotic exit [35, 36]. To determine at which stage of the cell cycle SIK2 inhibition impacts *FANCA* ^−/−^ cells, we analyzed cell cycle profiles of *FANCA* ^−/−^ vs *FANCA* ^+^ cells following ARN‐3236 exposure. ARN‐3236 treatment caused a small, but significant, increase in the percentage of *FANCA* ^+^ cells with 4N DNA content. This ARN‐3236‐induced 4N accumulation was dramatically increased in *FANCA* ^−/−^ cells (Fig. 4A,B). Phospho‐histone H3 (Ser10), a mitotic marker, was significantly decreased in cells treated with ARN‐3236 (Fig. 4C), suggesting that cells were blocked from entering mitosis by SIK2 inhibition. Notably, phospho‐H3 was more significantly reduced in *FANCA* ^−/−^ cells (Fig. 4C). Consistently, live‐cell imaging revealed that SIK2 inhibition reduced the number of cells entering mitosis, with this number being more significantly reduced in *FANCA* ^−/−^ fibroblasts and *FANCA*‐knockdown HeLa cells relative to their respective *FANCA* ^+^ counterparts (Fig. 4D,E). Of ARN‐3236‐treated HeLa cells that did enter mitosis, quantification of time from cell rounding to flattening revealed that mitotic exit was delayed, with this delay being significantly prolonged in *FANCA*‐knockdown cells (Fig. 4F,G).

### 3.3 FANCA and SIK2 colocalize at mitotic structures

**Fig. 5:**
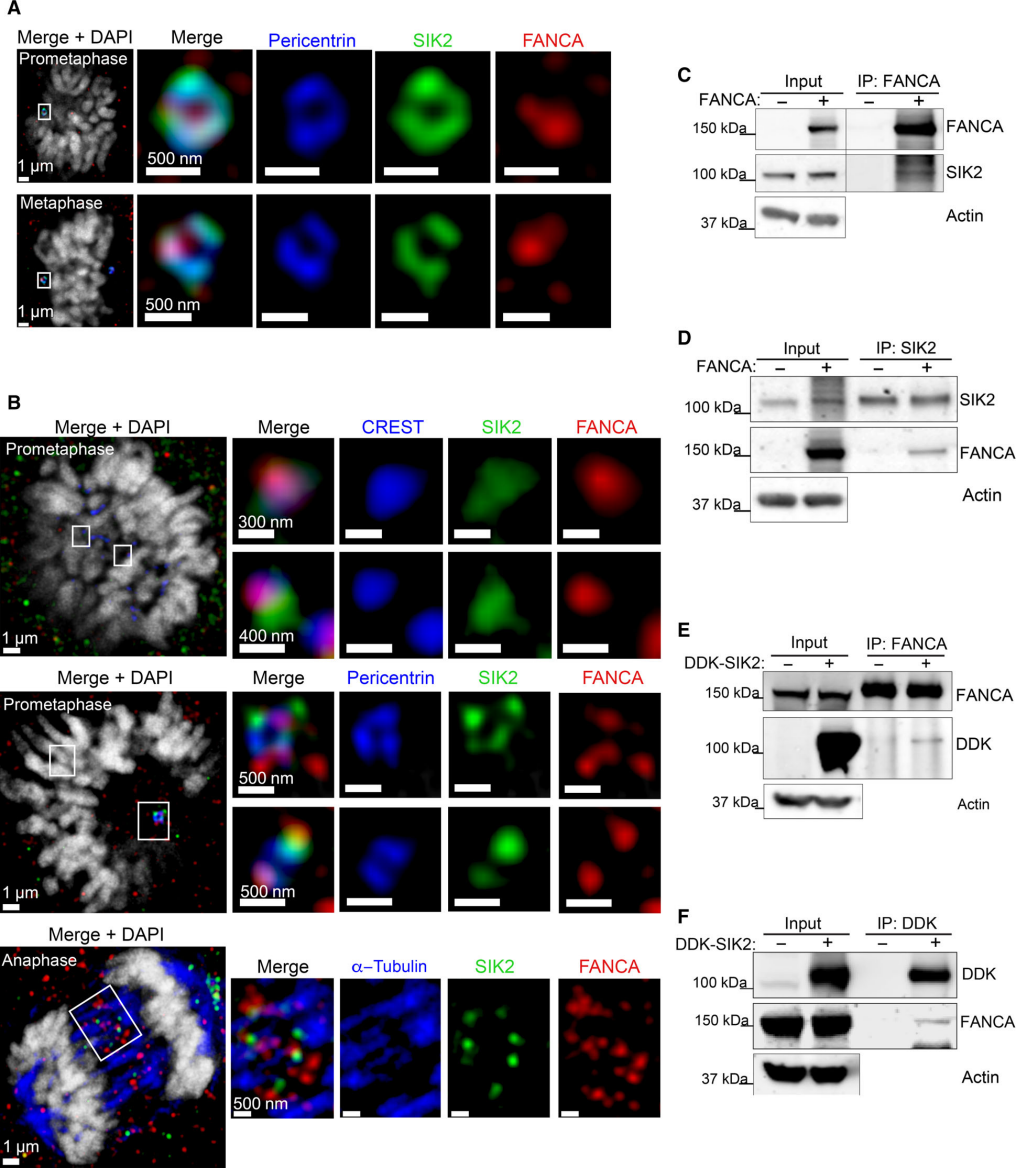
SIK2 is a novel FANCA interactor. (A) Colocalization of FANCA and SIK2 to the centrosome. FANCA (red) and SIK2 (green) shown colocalizing with pericentrin (blue), which marks the centrosome, in *FANC*A‐corrected fibroblasts (*FANCA* ^+^). Scale bars for left panels represent 1 μm. Scale bars for enlargements of centrosomes represent 500 nm. (B) Colocalization of FANCA (red) and SIK2 (green) at the centrosome, kinetochore, and central spindle in HeLa cells. CREST (blue) and tubulin (blue) were used to mark the kinetochore and mitotic spindle, respectively. Scale bars for left panels represent 1 μm. For panels showing enlargements of centrosomes, scale bars represent 300–500 nm (specified in panel). (C) Immunoprecipitation of FANCA in *FANC*A‐corrected patient fibroblasts. (D) Immunoprecipitation of SIK2 in *FANC*A‐corrected patient fibroblasts. (E) Immunoprecipitation of FANCA in HEK293T cells overexpressing SIK2 (DDK‐SIK2). (F) Immunoprecipitation of SIK2 in HEK293T cells overexpressing SIK2 (DDK‐SIK2).

To determine whether FANCA and SIK2 have a functional relationship, we examined their immunolocalization during mitosis. Individually, both FANCA and SIK2 have been found to localize to the centrosome [4, 35]. Using immunofluorescence, we found that FANCA colocalized with SIK2 at the centrosome during prometaphase and metaphase (Fig. 5A). We also observed previously unreported localization of SIK2 to kinetochores and the central spindle. Interestingly, kinetochore‐associated SIK2 colocalized with FANCA, and central spindle‐associated SIK2 partially overlapped with FANCA (Fig. 5B). Localization of FANCA and SIK2 to mitotic kinetochores and central spindles was validated by three unique antibodies per protein (Figs S4, S6 and S7).

To determine whether FANCA interacts with SIK2, we immunoprecipitated FANCA from *FANCA* ^+^ patient fibroblasts and HEK293T cells expressing DDK‐tagged SIK2. In both conditions, SIK2 co‐immunoprecipitated with FANCA (Fig. 5C,E and Fig. S2). These results were confirmed by reciprocal co‐immunoprecipitations (Fig. 5D,F). Together, these results provide evidence of a physical interaction between FANCA and SIK2.

### 3.4 Phosphorylation of SIK2 at serine‐358 is decreased in *FANCA*‐deficient cells

**Fig. 6:**
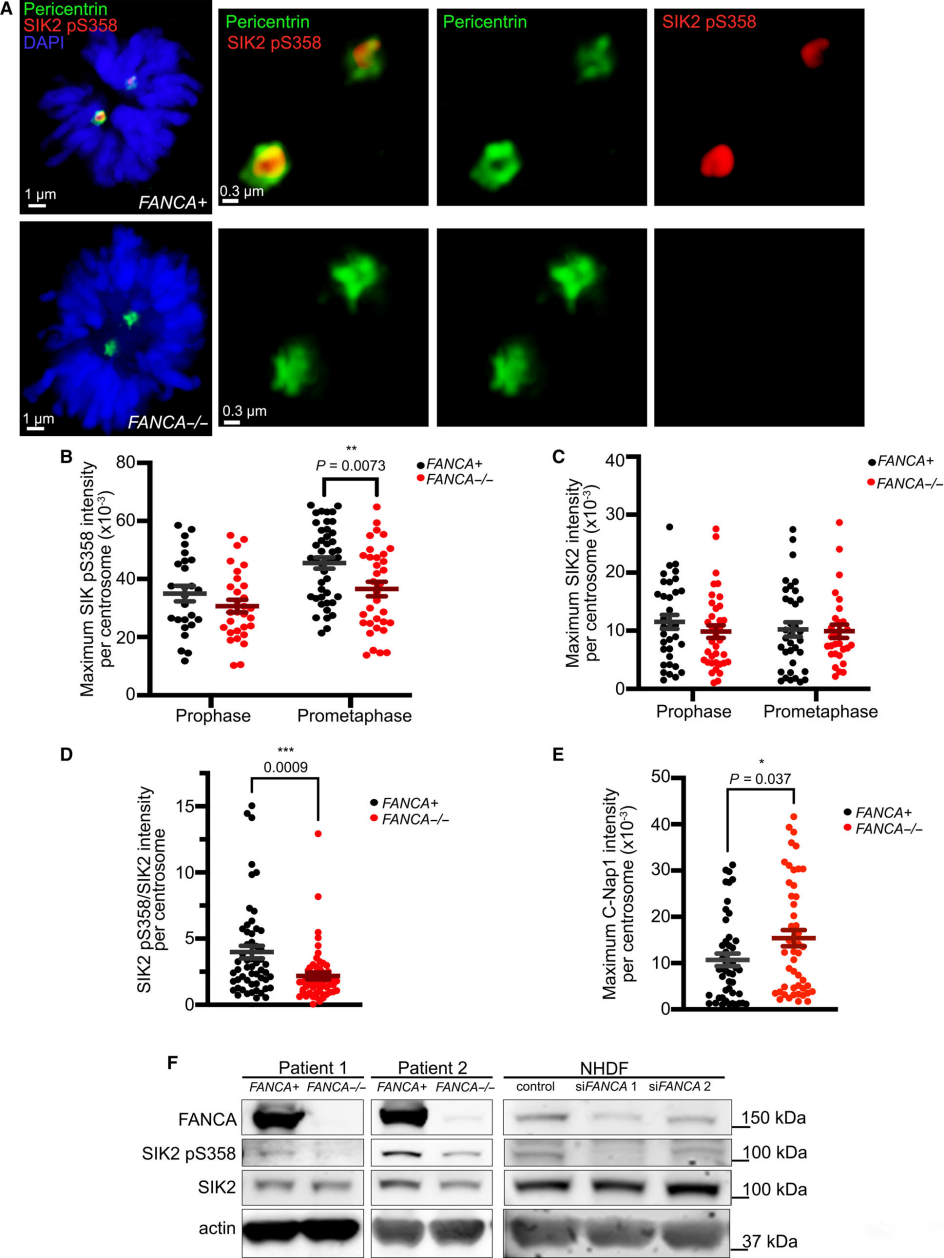
*FANCA* deficiency impairs SIK2 activity. (A) Localization of SIK2 pS358 to centrosome in *FANCA* ^+^ (upper panel) and *FANCA* ^−/−^ (lower panel) patient fibroblasts. Pericentrin (green), SIK2 pS358 (red), and DAPI (blue). Scale bars for left panels represent 1nm. For panels showing enlargements of centrosomes, scale bars represent 0.3 μm. (B) Quantification of SIK2 pS358 intensity on centrosomes. The intensity was compared between *FANCA* ^+^ and *FANCA* ^−/−^ fibroblasts in prophase and prometaphase. Plot shows data pooled from three independent experiments (mean ± SEM; *P* value was calculated by one‐way ANOVA). For *FANCA* ^+^ vs *FANCA* ^−/−^ prometaphase cells, *P* = 0.0073. At least 29 centrosomes were analyzed per condition. (C) Quantification of SIK2 intensity on centrosomes of *FANCA* ^−/−^ primary fibroblasts and its corrected counterpart. Plot shows data pooled from three independent experiments (mean ± SEM; *P* value was calculated by one‐way ANOVA). At least 29 centrosomes were analyzed per condition. (D) Quantification of the intensity ratio SIK2 pS358/ SIK2 in *FANCA* ^−/−^ primary fibroblast prometaphase cells. The ratio plot shows representative data pooled from two independent experiments (mean ± SEM; *P* = 0.0009, as calculated by one‐way ANOVA). At least 28 centrosomes were analyzed per condition. (E) Quantification of C‐Nap1 intensity on centrosomes of *FANCA* ^−/−^ primary fibroblast and its corrected counterpart. Plot shows data pooled from three independent experiments (mean ± SEM; *P* = 0.037, as calculated by unpaired *t*‐test). At least 47 centrosomes were analyzed per condition. (F) Representative western blot showing reduced levels of SIK2 pS358 in *FANCA*‐deficient cells. Two different pairs of primary *FANCA*‐deficient patient fibroblasts and their gene‐corrected counterparts were used. NHDF cells with siRNA‐mediated *FANCA* knockdown (90% knockdown in si*FANCA*1 and 57% knockdown in si*FANCA*2) exhibited lower levels of SIK2 pS358 (30–44% less) when compared to nontargeting control siRNA‐transfected NHDF cells, while total SIK2 levels were unchanged. The quantification was average of two independent experiments.

We next investigated the effect of FANCA loss on SIK2 function. SIK2 is known to localize to centrosomes during early mitosis to facilitate bipolar spindle assembly [35]. We therefore utilized quantitative immunofluorescence to examine SIK2 recruitment to mitotic centrosomes in *FANCA* ^−/−^ cells (Fig. 6A). Total SIK2 accumulated at prophase and prometaphase centrosomes with equal intensity in *FANCA* ^−/−^ and *FANCA* ^+^ fibroblasts (Fig. 6C). However, SIK2 pS358 intensity was significantly reduced in *FANCA* ^−/−^prometaphase centrosomes relative to *FANCA* ^+^ counterparts (Fig. 6A,B). Normalizing the SIK2 pS358 intensity to total SIK2 intensity per centrosome also indicated that *FANCA* ^−/−^ fibroblasts had significantly lower SIK2 pS358 to total SIK2 ratio in prometaphase centrosomes (Fig. 6D). Consistent with these findings, western blot analysis of whole cell lysates prepared from two FA patient‐derived *FANCA* ^−/−^ fibroblasts vs isogenic *FANCA*‐complemented fibroblasts revealed reduced levels of SIK2 pS358 in *FANCA*‐deficient cells, while total SIK2 levels were unaffected by *FANCA* status (Fig. 6F). Likewise, SIK2 pS358 levels were significantly reduced in NHDFs transfected with *FANCA*‐targeting siRNAs relative to cells transfected with nontargeting siRNA, while total SIK2 levels were unchanged (Fig. 6F, right panel). To further confirm reduction of SIK2 activity in *FANCA* ^−/−^ fibroblasts, we quantified the immunofluorescence of C‐Nap1, a downstream target of SIK2, which is displaced from the centrosome after phosphorylation by SIK2 [35]. Thus, increased C‐Nap1 intensity at the centrosome indicates reduced SIK2 activity. We found that *FANCA* ^−/−^ fibroblasts exhibited significantly higher C‐Nap1 intensity on prometaphase centrosomes than *FANCA* ^+^ fibroblasts, further indicating that *FANCA* deficiency results in impaired SIK2 activity (Fig. 6E and Fig. S3C).

### 3.5 SIK2 pS358 and FANCA exhibit dynamic patterns of colocalization throughout mitosis

**Fig. 7:**
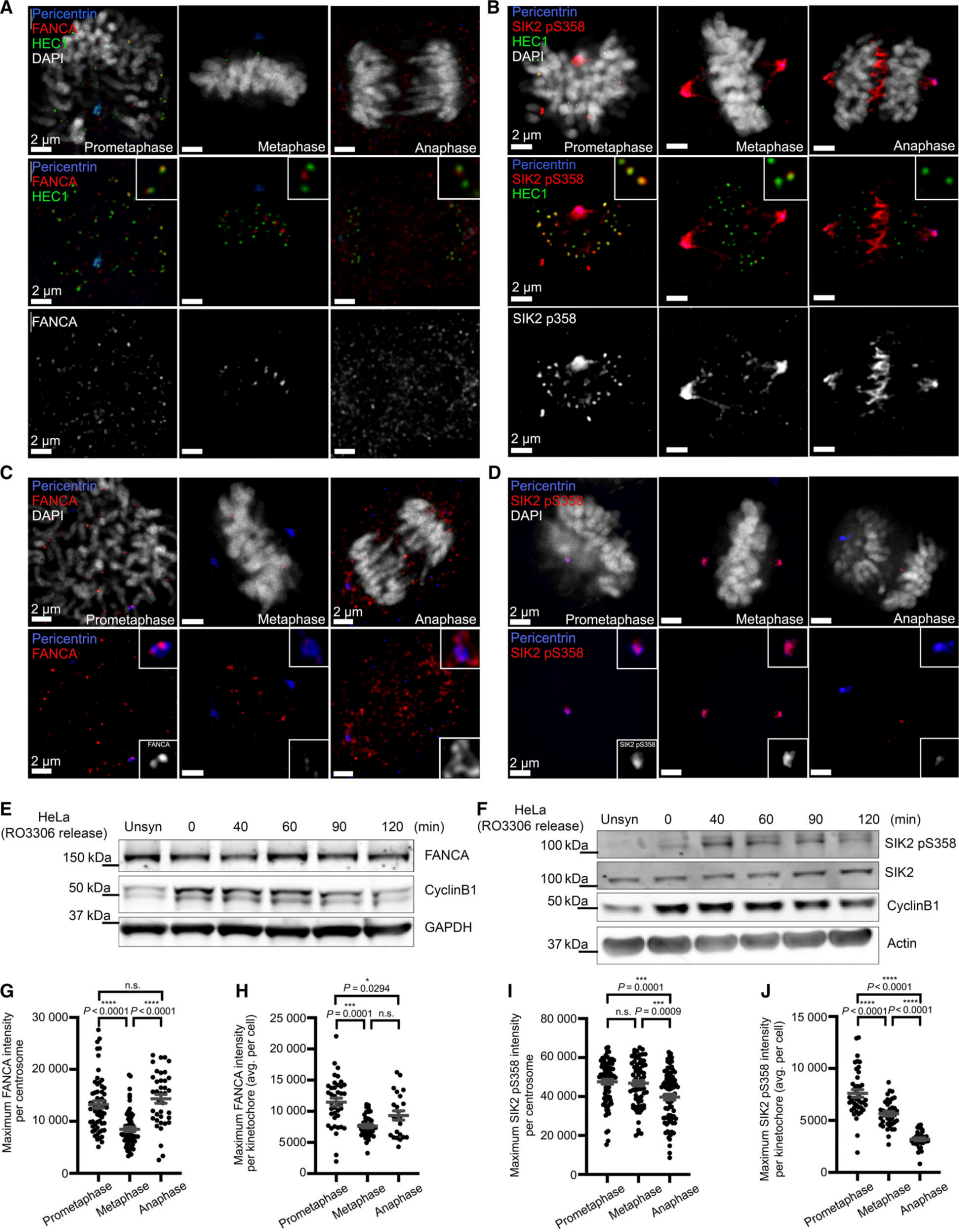
Dynamic changes in FANCA and SIK2 pS358 localization during mitosis. (A, C) Localization of FANCA on kinetochores (A) and centrosomes (C) in HeLa cells throughout mitosis. FANCA (red), pericentrin (blue), HEC1 (green), and DAPI (white). All scale bars represent 2 μm. (B, D) Localization of SIK2 pS358 on kinetochores (B) and centrosomes (D) in HeLa cells throughout mitosis. SIK2 pS358 (red), pericentrin (blue), HEC1 (green), and DAPI (white). All scale bars represent 2 μm. (E, F) Representative western blots showing the expression of FANCA (E) and SIK2 pS358 (F) in HeLa cells throughout mitosis. (G) Quantification of FANCA intensity per centrosome (*n* ≥ 38 centrosomes per stage). Plots show mean ± SEM of data pooled from two independent experiments. *P* values were calculated by one‐way ANOVA. For prometaphase vs metaphase, *P* < 0.0001. For metaphase vs anaphase, *P* < 0.0001. (H) Quantification of FANCA intensity per kinetochore (*n* ≥ 23 cells per stage). Plots show mean ± SEM of data pooled from two independent experiments. *P* values were calculated by one‐way ANOVA. For prometaphase vs metaphase, *P* = 0001. For prometaphase vs anaphase, *P* = 0.0294. (I) Quantification of SIK2 pS358 intensity per centrosome (*n* ≥ 73 centrosomes per stage). Plots show mean ± SEM of data pooled from two independent experiments. At least 73 centrosomes per mitotic stage were analyzed. *P* values were calculated by one‐way ANOVA. For prometaphase vs anaphase, *P* = 0.0001. For metaphase vs anaphase, *P* = 0.0009. (J) Quantification of SIK2 pS358 intensity per kinetochore (*n* ≥ 37 cells per stage). Plots show mean ± SEM of data pooled from two independent experiments. At least 73 centrosomes per mitotic stage were analyzed. *P* values were calculated by one‐way ANOVA. For all comparisons, *P* < 0.0001.

To further characterize mitotic functions of SIK2 and explore its functional relationship with FANCA, we first assessed whether their expression was cell cycle‐dependent. Total SIK2 and SIK2 pS358 levels were assessed relative to the mitotic marker, cyclin B1. Western blot analysis of thymidine block‐synchronized HeLa cells revealed that while total SIK2 levels remained constant, an increase in SIK2 S358 phosphorylation occurred simultaneously with the surge in cyclin B1 during mitosis (Fig. S3D). Likewise, we observed increased levels of SIK2 pS358 in HeLa cells arrested in mitosis by treatment with nocodazole and taxol relative to untreated cells (Fig. S3E). While SIK2 pS358 demonstrated an increase in expression during mitosis, FANCA expression remained constant throughout the cell cycle (Fig. S3F). Additionally, western blot analysis demonstrated that the expression levels of FANCA and SIK2 pS358 were steady over the course of mitosis (Fig. 7E,F).

Next, we examined localization of SIK2 pS358 and FANCA during mitosis *via* immunofluorescence in HeLa cells. FANCA associated with the centrosomes and kinetochores throughout mitosis, with intensity varying from prometaphase to anaphase (Fig. 7A,C). Similarly, we also observed localization of SIK2 pS358 at centrosomes and kinetochores throughout prometaphase to anaphase with varying intensities (Fig. 7B,D). Quantification of FANCA localization at centrosomes (Fig. 7G) showed decreased intensity during metaphase, and its intensity at kinetochores (Fig. 7H) also decreased from prometaphase toward metaphase and anaphase. SIK2 pS358 intensity at centrosomes decreased in anaphase compared to prometaphase and metaphase (Fig. 7I), whereas the intensity at kinetochores decreased progressively from prometaphase to metaphase and anaphase (Fig. 7J). In addition to the individual, dynamic changes in SIK2 pS358 and FANCA at these mitotic structures, we also observed through co‐immunostaining that a portion of FANCA colocalized with SIK2 pS358 at mitotic centrosomes, kinetochores, and the central spindle (Fig. S4A). Of note, kinetochore association of both FANCA and SIK2 pS358 peaked at prometaphase, the stage of maximal SAC activity (Fig. 7H,J). These findings demonstrate novel subcellular localization patterns for FANCA and SIK2 and suggest stage‐specific functions at these mitotic structures.

### 3.6 SIK2 inhibition induces polynucleation

**Fig. 8:**
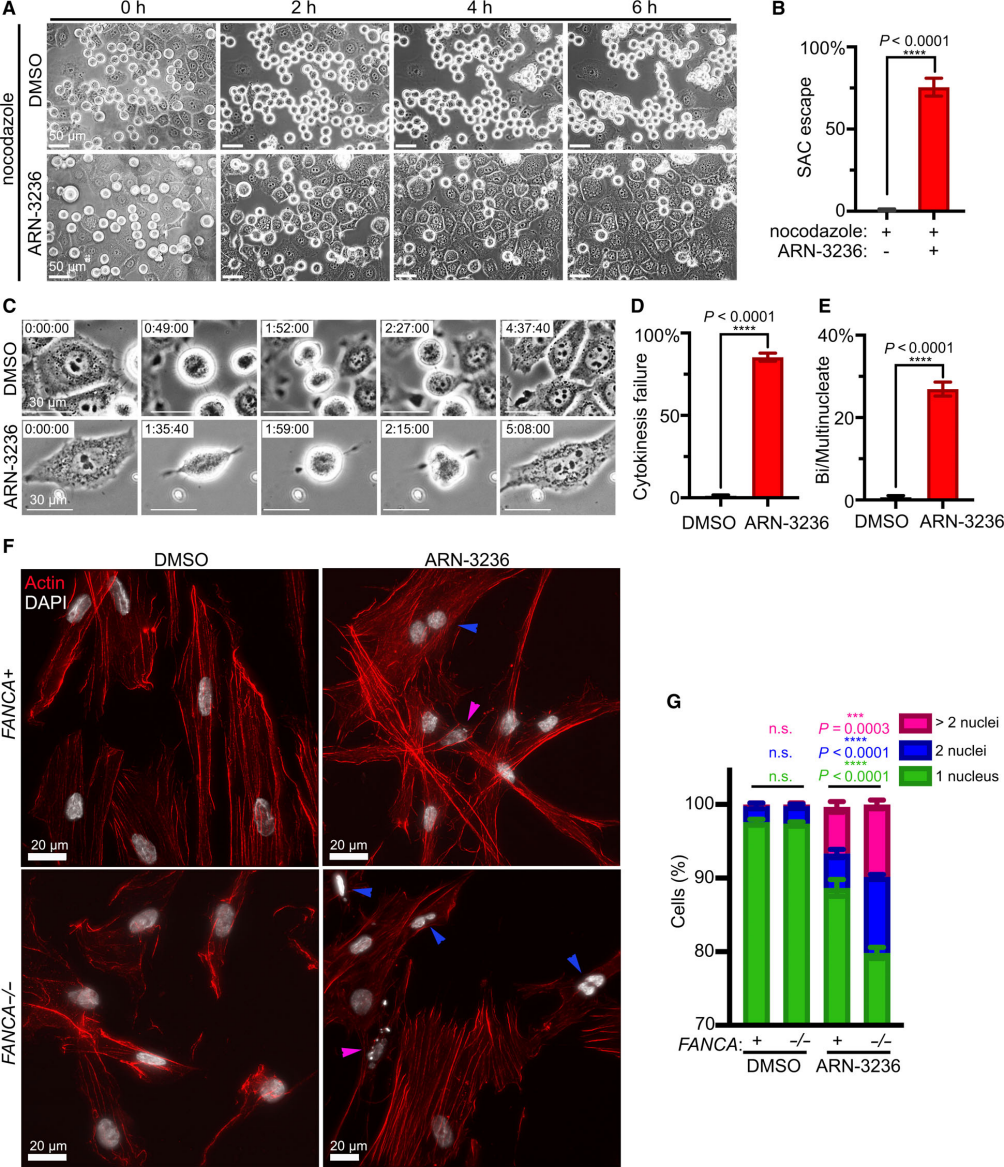
SIK2 inhibition impairs SAC function and cytokinesis. (A) Representative live imaging stills of HeLa cells that were synchronized with RO3306 (9 μm) for 20 h, released, and arrested in mitosis with nocodazole (150 nm) for 4 h prior to the addition of ARN‐3236 (1 μm). The mitotic cells were then imaged for 6 h following ARN‐3236 treatment. Scale bars represent 50 μm. (B) Quantification of the percentage of cells escaping from nocodazole‐induced mitotic arrest. Plot represents data pooled from three independent experiments. At least 35 cells per condition were analyzed for each experiment. *P* value was calculated by unpaired *t*‐test (*P* < 0.0001). (C) Live imaging stills of HeLa cells treated with ARN‐3236 (1 μm) or DMSO. Scale bars represent 30 μm. (D) Quantification of the percentage of HeLa cells failing to divide following mitosis during a 24 h time course. Plot shows mean ± SEM of data pooled from two independent experiments. At least 49 cells per condition were analyzed for each experiment. *P* value was calculated by unpaired *t*‐test (*P* < 0.0001). (E) Quantification of the percentage of bi‐/multinucleated HeLa cells formed during a 24 h time course. Plot shows mean ± SEM of data pooled from two independent experiments. At least 49 cells per condition were analyzed for each experiment. *P* value was calculated by unpaired *t*‐test (*P* < 0.0001). (F) *FANCA* ^−/−^ patient fibroblasts and *FANCA* ^+^ counterparts were synchronized by using RO3306 (9 μm) for 20 h. The fibroblasts were then released and treated with DMSO or ARN‐3236 (1 μm) for 4 h. The fibroblasts were stained with phalloidin (red) and DAPI (grayscale). Binucleated (blue arrows) and multinucleated cells (pink arrows) were counted. Scale bars represent 20 μm. (G) Quantification of bi‐/multinucleated cells formed after ARN‐3236 treatment of patient fibroblasts. Plot shows data pooled from three independent experiments. At least 99 cells per condition were analyzed for each experiment. *P* value was calculated by two‐way ANOVA with Tukey's multiple comparisons ad hoc test. For *FANCA* ^+^ vs *FANCA* ^−/−^ cells treated with ARN‐3236, cells with 1 nucleus, 2 nuclei, or > 2 nuclei, *P* values were < 0.0001, < 0.0001, and 0.0003, respectively.

Given our observation that SIK2 pS358 localizes to the kinetochore, we wondered whether SIK2 has a function in the SAC [46]. To address this question, we synchronized HeLa cells by arresting in G2 *via* treatment with the CDK1‐inhibitor RO3306 and then released the cells into nocodazole‐containing media to induce SAC arrest (Fig. 8A). After 4 h of nocodazole treatment, cells were additionally treated with ARN‐3236 (or DMSO control) and observed *via* time‐lapse imaging. While mitotic arrest was maintained in control cells, ARN‐3236 induced SAC escape, resulting in severe multinucleation (Fig. 8A,B). This finding suggests that SIK2 activity is required for SAC function. In support of this notion, western blot analysis demonstrated that ARN‐3236 treatment of nocodazole‐arrested cells decreased levels of cyclin B1, as well as SAC proteins BUB1 and Aurora B, indicating silencing of the SAC (Fig. S4B).

Another potential source of ARN‐3236‐induced polyploidy is cytokinesis failure, which has previously been observed in SIK2‐depleted cells [35, 36]. Through live imaging, we monitored mitotic progression of HeLa cells treated with ARN‐3236 and observed that these cells initiated cleavage furrow ingression but failed to trigger abscission (Fig. 8C,D). Such cytokinesis failure resulted in formation of bi‐ or multinucleated cells (Fig. 8E).

To determine whether ARN‐3236‐induced polynucleation was enhanced by *FANCA* deficiency, patient fibroblasts were arrested in G2 using RO3306, then released into ARN‐3236‐ or DMSO‐containing media, and allowed to proceed through mitosis. After sufficient time to complete cell division, fibroblasts were fixed, and nucleation was assessed *via* fluorescence microscopy. While *FANCA* ^+^ and *FANCA* ^−/−^ fibroblasts exhibited a striking increase in bi‐ and multinucleated cells upon treatment with ARN‐3236, both forms of polynucleation were significantly higher in *FANCA* ^−/−^ cells (Fig. 8F,G).

### 3.7 *SIK2* is a synthetic lethal target of multiple FA genes

**Fig. 9:**
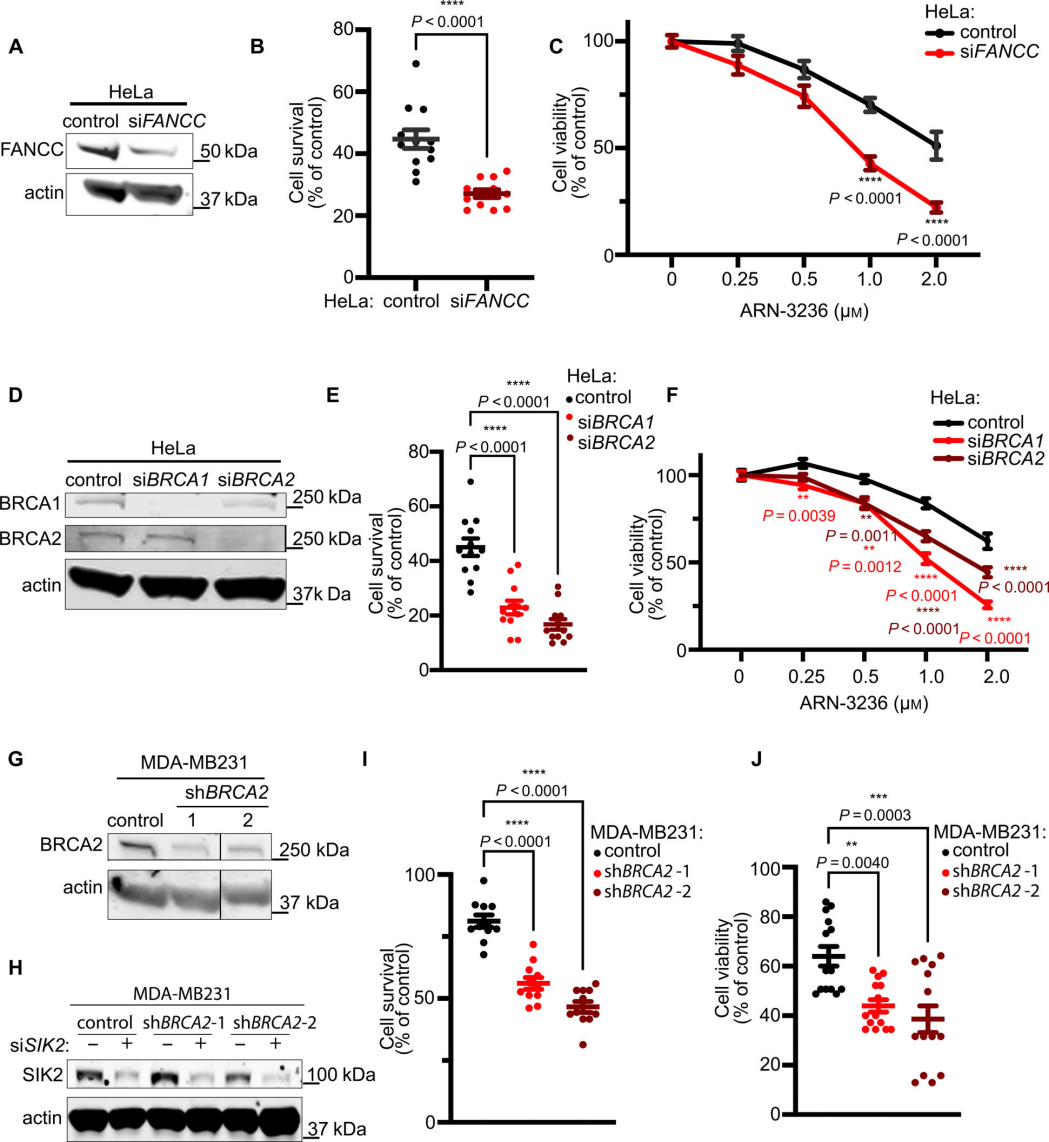
Multiple FA pathway genes are synthetic lethal targets of *SIK2*. (A) Representative western blot showing FANCC expression in HeLa cells transfected with nontargeting vs *FANCC*‐targeting siRNA. An average 59% decrease in FANCC was observed over three independent experiments. (B) Cell survival of HeLa‐si*FANCC* cells treated with ARN‐3236. The cell survival was measured by trypan blue exclusion after 48 h treatment and plot shows mean ± SEM of data pooled from three independent experiments. *P* values were calculated by unpaired *t*‐test (*P* < 0.0001). (C) Cell viability of HeLa‐si*FANCC* cells treated with ARN‐3236. CellTiter‐Glo assay was used to measure the cell viability after 72 h treatment and the plot shows mean ± SEM of data pooled from four independent experiments. *P* values were calculated by two‐way ANOVA with ad hoc Sidak's multiple comparison test. For control vs si*FANCC* HeLa cells treated with 1or 2 μm ARN‐3236, *P* < 0.0001. (D) Representative western blot showing BRCA1 and BRCA1 expression in HeLa cells transfected with nontargeting vs *BRCA1*‐ and *BRCA2*‐targeting siRNAs. BRCA1 and BRCA2 expression was reduced by averages of 77% and 89%, respectively, over three independent experiments. (E) Cell survival of HeLa‐si*BRCA1* and HeLa‐si*BRCA2* cells treated with ARN‐3236. The cell survival was measured by trypan blue exclusion after 48 h treatment. Plot shows mean ± SEM of data pooled from four independent experiments. *P* values were calculated by one‐way ANOVA with ad hoc Sidak's multiple comparison test. For both comparisons, *P* < 0.0001. (F) Cell viability HeLa‐si*BRCA1* and HeLa‐si*BRCA2* cells treated with ARN‐3236. CellTiter‐Glo (right) assay was used to measure the cell viability after 72 h treatment. Plot shows mean ± SEM of data pooled from three independent experiments. *P* values were calculated by two‐way ANOVA with ad hoc Tukey's multiple comparison test. For control vs si*BRCA1* HeLa cells treated with 0.25, 0.5, 1, or 2 μm ARN‐3236, *P* values equaled 0.0039, 0.0012, < 0.0001, and < 0.0001, respectively. For control vs si*BRCA2* HeLa cells treated with 0.5, 1, or 2 μm ARN‐3236, *P* values equaled 0.0011, < 0.0001, and < 0.0001, respectively. (G) Representative western blot demonstrating reduced BRCA2 expression in two stable sh*BRCA2* knockdown clones of breast cancer cell line MDA‐MB231 vs MDA‐MB231 stably transduced with nontargeting shRNA. An average 47% and 36% decrease in BRCA2 was observed in sh*BRCA2*‐*1* and sh*BRCA2*‐*2,* respectively, from two independent experiments. (H) Representative western blot demonstrating reduced SIK2 expression in *BRCA2* knockdown MDA‐MB231 cells transfected with si*SIK2*. SIK2 levels were decreased by averages of 72%, 64%, and 67% in control MDA‐MBA231, MDA‐MB231‐sh*BRCA2*‐1, and MDA‐MB231‐sh*BRCA2*‐2, respectively, over three independent experiments. (I) Cell survival of MDA‐MB231‐sh*BRCA2* cells transfected with *SIK2*‐targeting siRNA vs nontargeting siRNA. The cell survival was measured by trypan blue exclusion 48 h after transfection. *P* values were calculated by one‐way ANOVA with ad hoc Tukey's multiple comparison test. For control MDA‐MB231 vs both sh*BRCA2*‐1 and *shBRCA2*‐2 MDA‐MB231 cells, *P* < 0.0001. (J) Cell viability of MDA‐MB231‐sh*BRCA2* cells transfected with si*SIK2* vs nontargeting siRNA. The cell viability was measured by MTT assay. Plot shows mean ± SEM of data pooled from three independent experiments. *P* values were calculated by one‐way ANOVA with ad hoc Tukey's multiple comparison test. For control vs sh*BRCA2*‐1 MDA‐MB231 cells, *P* = 0.0040. For control vs *shBRCA2*‐2 MDA‐MB231 cells, *P* = 0.0003.

In addition to FANCA, other FA family members, such as FANCC, BRCA1, and BRCA2, have been shown to participate in mitosis [4, 47, 48, 49]. We therefore reasoned that SIK2 inhibition may be synthetic lethal to cells deficient in other FA genes. To address this possibility, we knocked down *FANCC*, *BRCA1,* and *BRCA2* in HeLa cells using gene‐specific siRNAs (Fig. 9A,D). We then tested their responses to SIK2 inhibition and showed that depletion of *FANCC, BRCA1,* or *BRCA2* resulted in hypersensitivity to ARN‐3236 treatment compared to the nontargeting siRNA control (Fig. 9B,C,E,F). Because loss of *BRCA2* is associated with susceptibility to breast cancer, we generated two clonal cell lines with stable *BRCA2* knockdown in the breast cancer cell line MDA‐MB231to assess synthetic lethality with *SIK2* in a relevant cancer type. We found that siRNA‐mediated depletion of *SIK2* significantly reduced cell survival and viability of both MDA‐MB231‐sh*BRCA2* cell lines compared to control MDA‐MB231 cells (Fig. 9G–J). These results suggest that *SIK2* is a synthetic lethal target of multiple FA pathway genes.

## 4 Discussion

Strict regulation of mitosis is essential to the preservation of genomic integrity during cell proliferation. Mitotic errors may result in chromosome misalignment, segregation defects, or faulty cell division, potentially culminating in genomic instability and subsequent malignancy [50]. FA pathway signaling has been reported to affect S phase progression through DNA damage response, but its direct participation in mitosis is less studied [51]. Previous studies demonstrated that individual FA proteins exhibit unique localization patterns during mitosis, which is suggestive of distinct mitotic functions [4]. FANCA, a fundamental member of the FA core complex, has also been shown to interact with non‐FA proteins in order to carry out DNA repair responses [52, 53]. FANCA was shown to affect centrosome integrity [3] and cytokinesis [54]. Both of these functions are critical to the progression and fidelity of mitosis; however, the underlying involvement of FANCA in these processes remains elusive. Underscoring the importance of FANCA function in mitosis, our kinome screen for synthetic lethal *FANCA* targets identified numerous candidates known to regulate mitosis. The spectrum of their functions span mitotic entry, SAC, and cytokinesis, strongly suggesting that mitotic processes are particularly vulnerable in FA pathway‐deficient cells. As a result, the simultaneous disruption of mitotic and FA signaling pathways may result in a more pronounced inhibition of cell growth. Here, we show that FANCA and SIK2 are interacting partners that colocalize at multiple mitotic structures. We found that phosphorylation of SIK2 S358, which correlates with SIK2 activity [45], is associated with cyclin B1 surge during mitotic progression and that FANCA is required for optimal SIK2 S538 phosphorylation at centrosomes. In addition, we demonstrate that SIK2 inhibition affects mitosis in multiple ways, not only blocking mitotic entry and impairing cytokinesis [35, 36, 37], but also through compromise of the SAC.

The segregation of sister chromatids during mitosis is an important process to ensure proper distribution of genetic materials among daughter cells [55]. During metaphase, the SAC acts to ensure that each chromatid is stably attached to microtubules emanating from opposite poles before proceeding to anaphase. The kinetochore is the prime apparatus for spindle attachment, upon which sensors and effectors for SAC regulation assemble [56, 57]. We have previously shown that the FA signaling pathway regulates SAC activity and that FA‐deficient cells exhibit a weakened SAC (Fig. S5) [4, 5]. Here, we showed that SIK2 and FANCA colocalize on several mitotic structures, including centrosomes, kinetochores, and the central spindle in a stage‐specific manner. Interestingly, the trend of both SIK2 pS358 and FANCA on kinetochores shows similar changes during the mitotic progression from high in prometaphase to low in anaphase, which is temporally consistent with the respective activation and satisfaction of the SAC. It is therefore conceivable that SIK2 and FANCA signaling pathways may have overlapping functions in the SAC and mitotic progression.

SIK2 is also known to play a role in cytokinesis [35, 36]. Cytokinesis initiates during anaphase when the sister chromosomes separate and the microtubules rearrange to form the central spindle [58]. The central spindle is crucial for determining the plane of cleavage furrow where ingression occurs [59, 60]. Aberrant central spindle arrangement impairs furrow induction and, subsequently, abscission [61, 62]. We showed that SIK2 pS358 is enriched at the central spindle, suggesting that SIK2 may play a functional role at this key structure. As SIK2 inhibition resulted in decreased Aurora B expression, it is also possible that SIK2 may indirectly regulate cytokinesis through Aurora B, which is known to regulate central spindle formation and dynamics [63, 64, 65]. Importantly, *FANCA* deficiency is also known to induce cytokinesis failure [8]. Thus, functional overlap of FANCA and SIK2 in these key steps of mitosis may be the basis of their synthetic lethal interaction.

Germline biallelic loss of FA genes is rare: The incident rate of FA is approximately one out of 100 000 births in the United States [66]. However, recent studies have shown that the frequency of heterozygous FA gene mutations in cancer is underestimated [67, 68]. Somatic FA gene mutations have been found in different types of non‐FA‐associated malignancies with remarkable frequency [69]. As mentioned above, somatic disruptions within the FA pathway are also present in 30% of cancers documented in the TCGA PanCancer Atlas Studies (Table 1) [26, 27, 70, 71]. Therefore, synthetic lethal targets of the FA pathway are broadly significant to precision therapies for the general population, as well as FA patients. Until now, synthetic lethal targeting of the FA pathway has mainly focused on its role in DDR, identifying targets such as ATM, CHK1, FEN1, and most famously PARP1 [41, 42, 72, 73, 74, 75]. Beyond their roles in interphase DDR, recent work has demonstrated that FANCA, FANCC, BRCA1, and BRCA2 also have important functions in mitosis [4, 5, 48]. However, little attention has been given to synthetic lethal interactions between the FA pathway and known mitotic regulatory proteins. Thus, the mitotic dysregulation associated with FA pathway deficiency represents a potentially unexploited therapeutic target. AML is one of the most common cancers to develop in FA patients, and recent studies demonstrated efficacy of the pan‐SIK inhibitor, YKL‐05‐099, in the suppression of AML in mouse models [69, 76]. These findings suggested a potential role for SIK2 inhibition in the setting of FA‐associated AML and thus warranted further examination [69]. Here, we demonstrate that loss of at least two FA core complex components (*FANCA* and *FANCC*) and two downstream FA genes (*BRCA1* and *BRCA2*) is synthetic lethal upon SIK2 inhibition. These findings provide support for further assessment of the therapeutic efficacy of SIK2 inhibition as a single agent or in combination for the treatment of FA‐associated malignancies.

## 5 Conclusions

Synergistic approaches for the treatment of FA‐induced cancers are a critical need to reduce the toxic burden of standard chemotherapies in FA patients. Synthetic lethal targeting of the FA pathway is also significant to novel precision therapy approaches for the general population, in which 30% of cancers bear FA pathway mutations. Through an unbiased kinome‐wide shRNA screen, this work identified *SIK2* as a synthetic lethal target of *FANCA*. Our findings suggest a model in which suboptimal SIK2 function in *FANCA* ^−/−^ cells contributes to cell cycle dysregulation and mitotic defects, with further weakening of the FANCA‐SIK2 signaling axis *via* depletion or inhibition of SIK2 causing synthetic lethality.

## Conflict of interest

The authors declare no conflict of interest.

## Author contributions

GN and KKC conceived the studies. KKC, ZAS, DME, AS, DKM, and YH performed the experiments. ESP and DWC supervised the project. RS, AS, and SDR performed pathway analysis. KKC, ZAS, AS, DKM, RS, ESP, and DWC prepared the manuscript.

### Peer Review

The peer review history for this article is available at https://publons.com/publon/10.1002/1878‐0261.13027.

## Supporting information


**Fig. S1**. Depletion or inhibition of SIK2 is synthetic lethal in FAKO HeLa cells. (A) Representative Western blot demonstrating reduced SIK2 pS358 levels in HeLa cells treated with ARN‐3236 (1.5 μm) vs DMSO control for 48 h. An average 43% decrease of SIK2 pS358 from three independent experiments was seen. (B) Representative Western blot demonstrating reduced AKT S473 phosphorylation in *FANCA*‐corrected patient fibroblasts (left panel) and HeLa cells (right panel) upon ARN‐3236 treatment (2 μm and 1.5 μm, respectively). The AKT pS473 was 48% less (average of three independent experiments) in ARN‐treated patient fibroblasts and was 37% less (average of two independent experiments) in ARN‐treated HeLa cells.

**Fig. S2**. SIK2 is co‐immunoprecipitates with FANCA. (A, B) Representative Western blots showing co‐immunoprecipitation of SIK2 (A) and FANCA (B) from *FANCA*‐corrected patient fibroblasts.

**Fig. S3**. Specificity of SIK2 pS358 antibody and expression of SIK2 pS358 during mitosis. (A) Total SIK2 and SIK2 pS358 antibodies colocalize at CREST foci, centrosomes, and central spindle. Scale bars represent 2 μm. (B) Phosphatase treatment of lysates from patient fibroblasts and HeLa cells abolishes SIK2 pS358 signal. (C) Immunofluorescence staining of C‐Nap1 to centrosome in *FANCA* ^+^ (upper panel) and *FANCA* ^−/−^ (lower panel) patient fibroblasts in prometaphase (pericentrin‐green, C‐Nap1‐red, DAPI‐blue). Scale bars represent 2 μm. (D) Increase in SIK2 pS358 expression after thymidine release corresponding to the increase in cyclin B1. Relative fold change in SIK2 pS358 was significantly higher at the peak of cyclin B1 surge than at the point of lowest cyclin B1. Mean ± SEM of three independent experiments were shown. *P* value was calculated by unpaired *t*‐test. (E) Representative Western blots showing seven‐fold and four‐fold increase, respectively in SIK2 phosphorylation in HeLa cells that were arrested in mitosis by 15‐h treatment with nocodazole (100 nm) or taxol (100 nm) relative to unsynchronized control. Three independent experiments were conducted. (F) Representative Western blots showing comparable FANCA expression in HeLa cells arrested in mitosis by 15‐h treatment with nocodazole (100 nm) or taxol (100 nm) relative to unsynchronized control. Three independent experiments were done.

**Fig. S4**. FANCA and SIK2 pS358 colocalize on kinetochores and central spindle. (A) Representative immunofluorescence images of HeLa throughout mitosis. FANCA is stained with a polyclonal goat antibody (R&D Systems) distinct from Fig. 6. Scale bars in top 3 panels represent 2 μm. Scale bars in bottom panel represent 1 μm. (B) Representative Western blot demonstrating reduced expression of SAC proteins in nocodazole‐arrested HeLa cells (150 nm) following exposure to ARN‐3236 (1 μm). The average changes in BuB1 and Aurora B expression after ARN‐3236 treatment were 40% and 50% less, respectively for both 2 h and 4 h. Three independent experiments were done.

**Fig. S5**. Loss of FANCA promotes escape from taxol‐induced SAC arrest. (A, B) Quantification of phospho‐H3 in *FANCA* ^−/−^ and gene‐corrected patient fibroblasts MNHN (A) and JRAST (B) treated with taxol (100 nm) for 18 h. For *FANCA* ^+^ vs *FANCA* ^−/−^ MNHN fibroblasts treated with 100 nm taxol, *P* = 0.003. For *FANCA* ^+^ vs *FANCA* ^−/−^ JRAST fibroblasts treated with 100 nm taxol, *P* < 0.0001.

**Fig. S6**. Validation of SIK2 localization. Representative immunofluorescence images show localization of SIK2 to mitotic centromeres (A, B) and central spindle (C, D) using with two unique antibodies (Abcam and Cell Signaling). Scale bars for whole cell images represent 1 μm. Scale bars for images of enlarged regions represent 500nm.

**Fig. S7**. Validation of FANCA localization. (A–C) Representative immunofluorescence images show the localization of FANCA to mitotic centromeres, spindle, and central spindle using an additional FANCA antibody (Rabbit polyclonal; Abcam). Scale bars for whole cell images represent 1 μm. Scale bars for images of enlarged regions represent 500 nm.

**Data Set S1** Supplementary Material.

## Data Availability

FASTQ files of shRNA clone sequencing from our kinome screen is openly available in the ArrayExpress database at https://www.ebi.ac.uk/arrayexpress/experiments/E‐MTAB‐9802 (accession number E‐MTAB‐9802) [40].
