# Supplementary figures and images for: SIK2 kinase synthetic lethality is driven by spindle assembly defects in FANCA‐deficient cells
Source: Mol Oncol. 2021 Jun 28;16(4):860–84. doi: 10.1002/1878-0261.13027 (PMC8847993; doi:10.1002/1878-0261.13027)

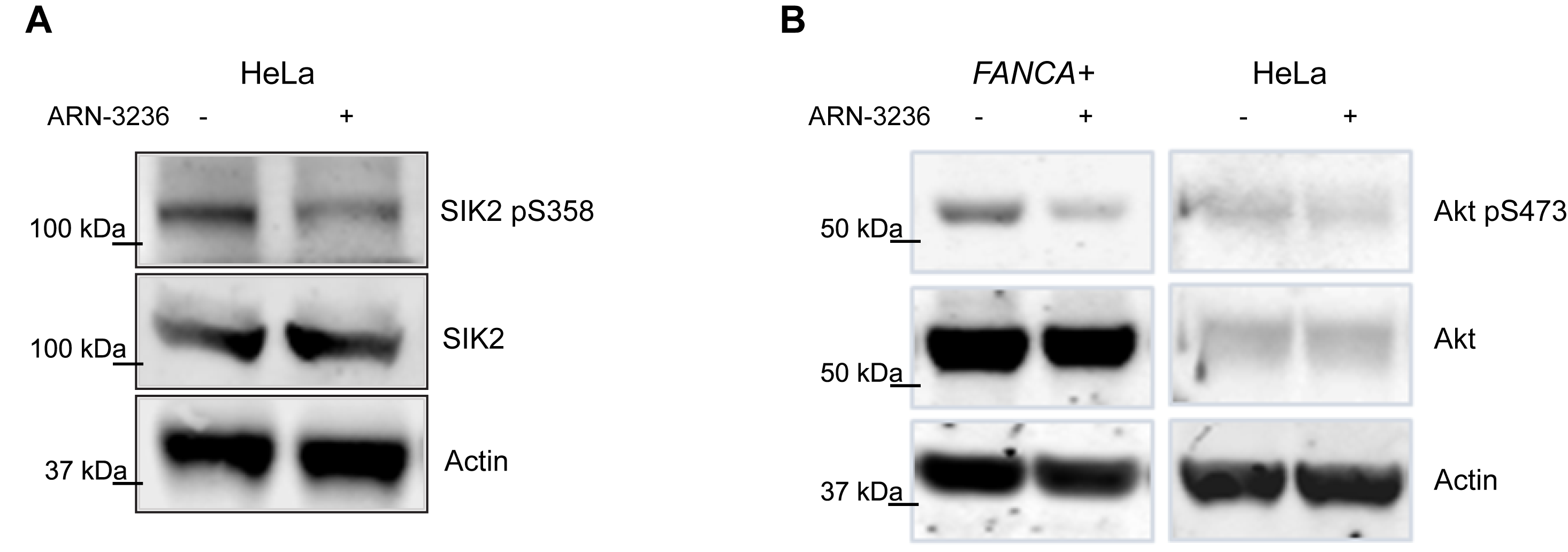

Supplement: Supplementary file 1 — Fig. S1. Depletion or inhibition of SIK2 is synthetic lethal in FAKO HeLa cells. (A) Representative Western blot demonstrating reduced SIK2 pS358 levels in HeLa cells treated with ARN‐3236 (1.5 μm) vs DMSO control for 48 h. An average 43% decrease of SIK2 pS358 from three independent experiments was seen. (B) Representative Western blot demonstrating reduced AKT S473 phosphorylation in FANCA‐corrected patient fibroblasts (left panel) and HeLa cells (right panel) upon ARN‐3236 treatment (2 μm and 1.5 μm, respectively). The AKT pS473 was 48% less (average of three independent experiments) in ARN‐treated patient fibroblasts and was 37% less (average of two independent experiments) in ARN‐treated HeLa cells. [file MOL2-16-860-s005.tif]

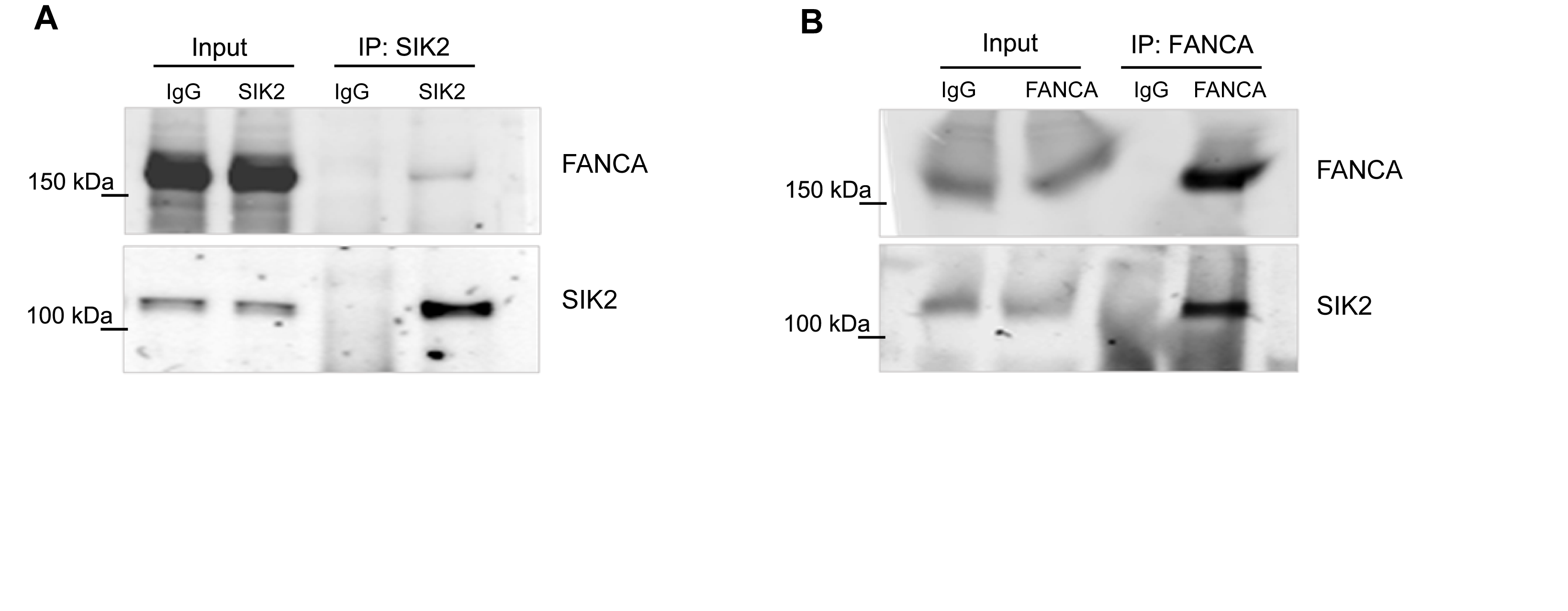

Supplement: Supplementary file 2 — Fig. S2. SIK2 is co‐immunoprecipitates with FANCA. (A, B) Representative Western blots showing co‐immunoprecipitation of SIK2 (A) and FANCA (B) from FANCA‐corrected patient fibroblasts. [file MOL2-16-860-s002.tif]

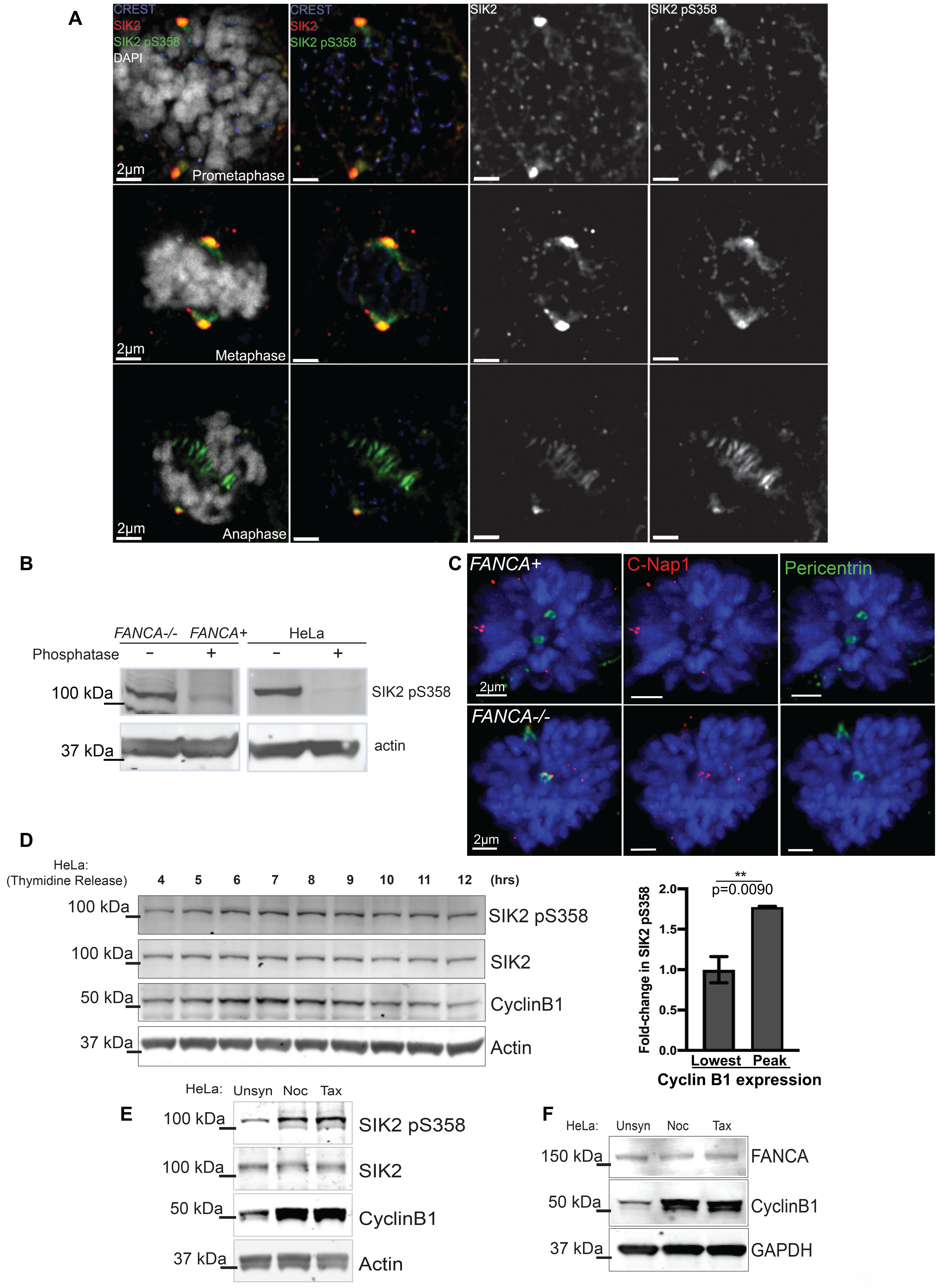

Supplement: Supplementary file 3 — Fig. S3. Specificity of SIK2 pS358 antibody and expression of SIK2 pS358 during mitosis. (A) Total SIK2 and SIK2 pS358 antibodies colocalize at CREST foci, centrosomes, and central spindle. Scale bars represent 2 μm. (B) Phosphatase treatment of lysates from patient fibroblasts and HeLa cells abolishes SIK2 pS358 signal. (C) Immunofluorescence staining of C‐Nap1 to centrosome in FANCA + (upper panel) and FANCA −/− (lower panel) patient fibroblasts in prometaphase (pericentrin‐green, C‐Nap1‐red, DAPI‐blue). Scale bars represent 2 μm. (D) Increase in SIK2 pS358 expression after thymidine release corresponding to the increase in cyclin B1. Relative fold change in SIK2 pS358 was significantly higher at the peak of cyclin B1 surge than at the point of lowest cyclin B1. Mean ± SEM of three independent experiments were shown. P value was calculated by unpaired t‐test. (E) Representative Western blots showing seven‐fold and four‐fold increase, respectively in SIK2 phosphorylation in HeLa cells that were arrested in mitosis by 15‐h treatment with nocodazole (100 nm) or taxol (100 nm) relative to unsynchronized control. Three independent experiments were conducted. (F) Representative Western blots showing comparable FANCA expression in HeLa cells arrested in mitosis by 15‐h treatment with nocodazole (100 nm) or taxol (100 nm) relative to unsynchronized control. Three independent experiments were done. [file MOL2-16-860-s006.tif]

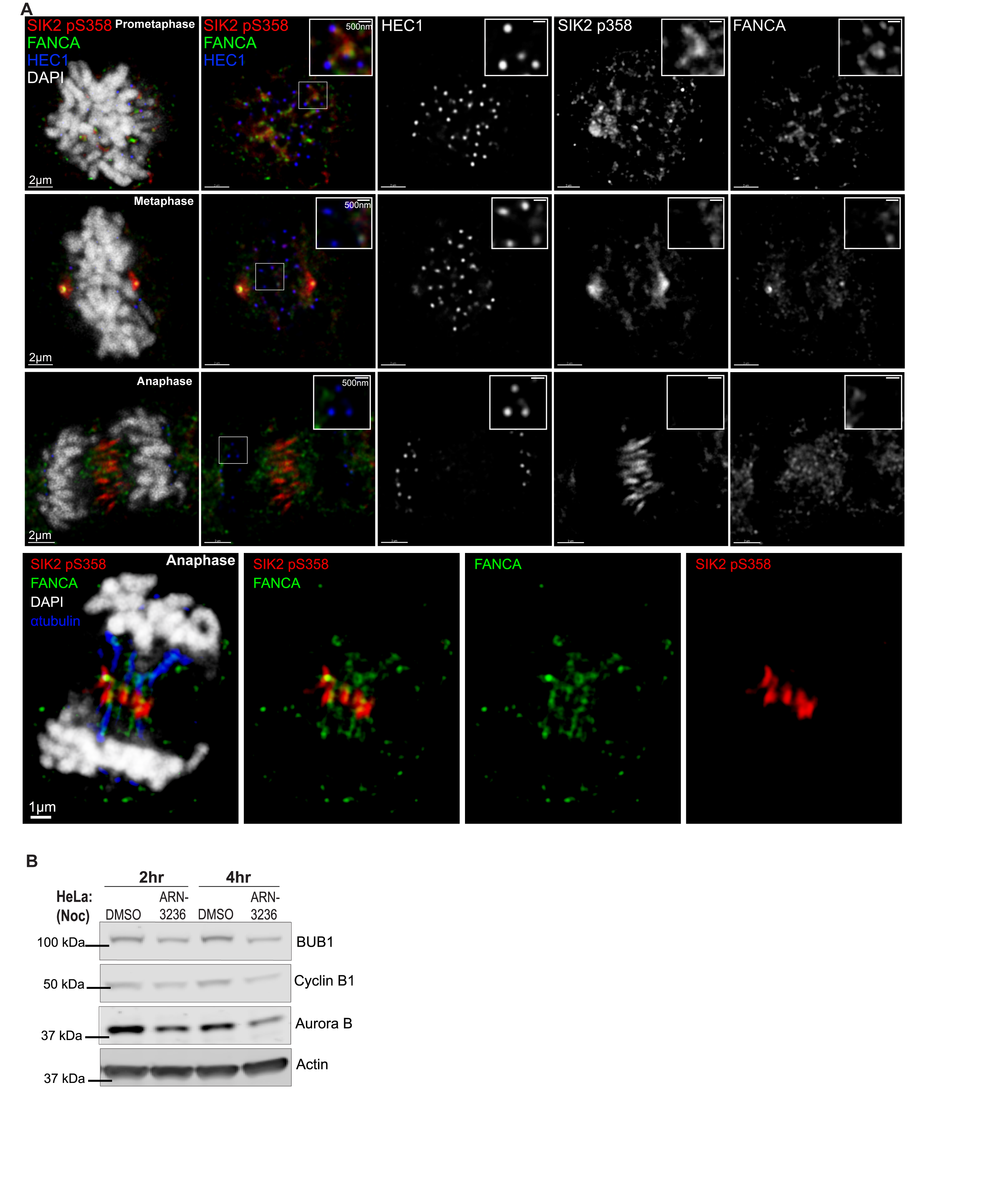

Supplement: Supplementary file 4 — Fig. S4. FANCA and SIK2 pS358 colocalize on kinetochores and central spindle. (A) Representative immunofluorescence images of HeLa throughout mitosis. FANCA is stained with a polyclonal goat antibody (R&D Systems) distinct from Fig. 6. Scale bars in top 3 panels represent 2 μm. Scale bars in bottom panel represent 1 μm. (B) Representative Western blot demonstrating reduced expression of SAC proteins in nocodazole‐arrested HeLa cells (150 nm) following exposure to ARN‐3236 (1 μm). The average changes in BuB1 and Aurora B expression after ARN‐3236 treatment were 40% and 50% less, respectively for both 2 h and 4 h. Three independent experiments were done. [file MOL2-16-860-s004.tif]

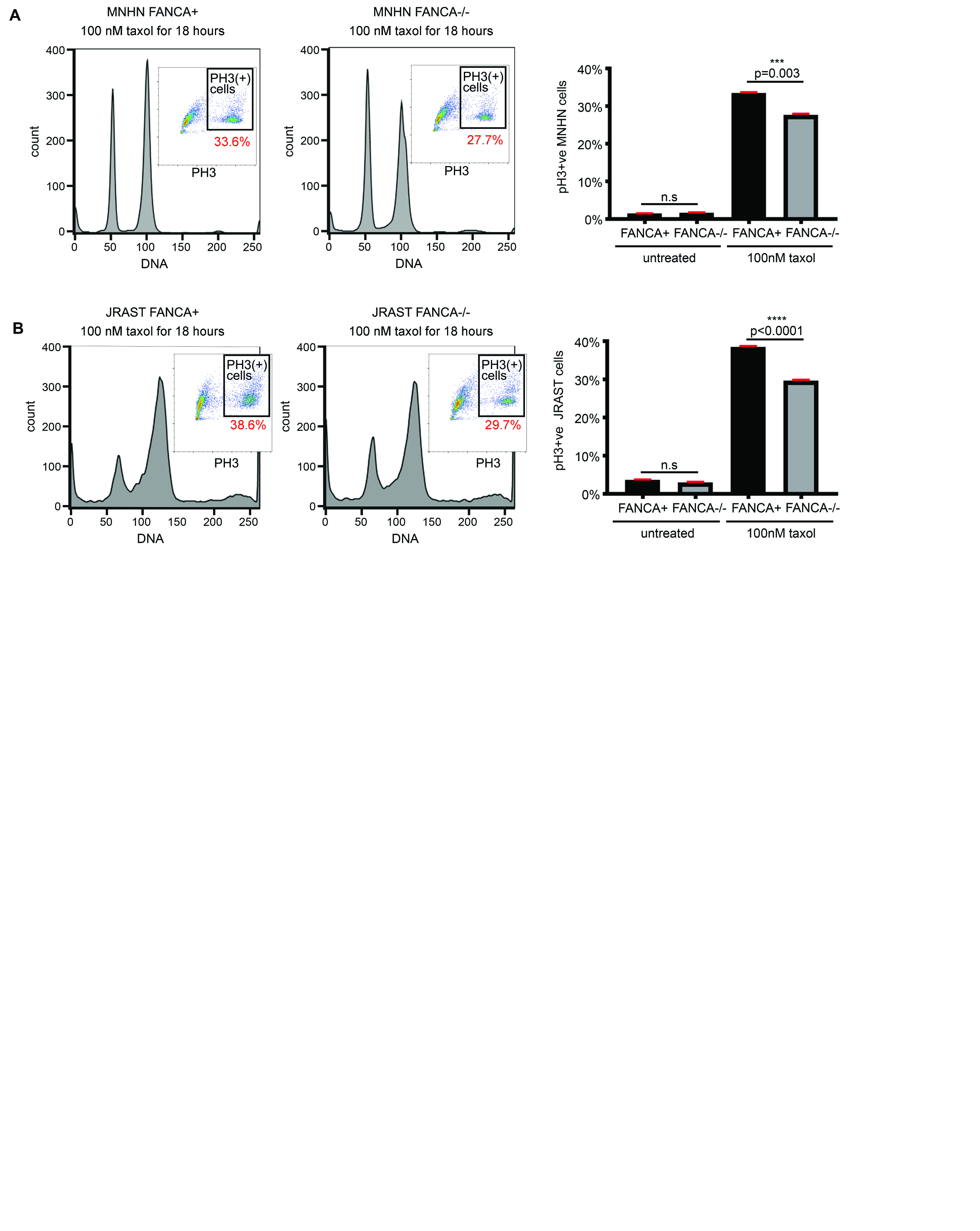

Supplement: Supplementary file 5 — Fig. S5. Loss of FANCA promotes escape from taxol‐induced SAC arrest. (A, B) Quantification of phospho‐H3 in FANCA −/− and gene‐corrected patient fibroblasts MNHN (A) and JRAST (B) treated with taxol (100 nm) for 18 h. For FANCA + vs FANCA −/− MNHN fibroblasts treated with 100 nm taxol, P = 0.003. For FANCA + vs FANCA −/− JRAST fibroblasts treated with 100 nm taxol, P < 0.0001. [file MOL2-16-860-s003.tif]

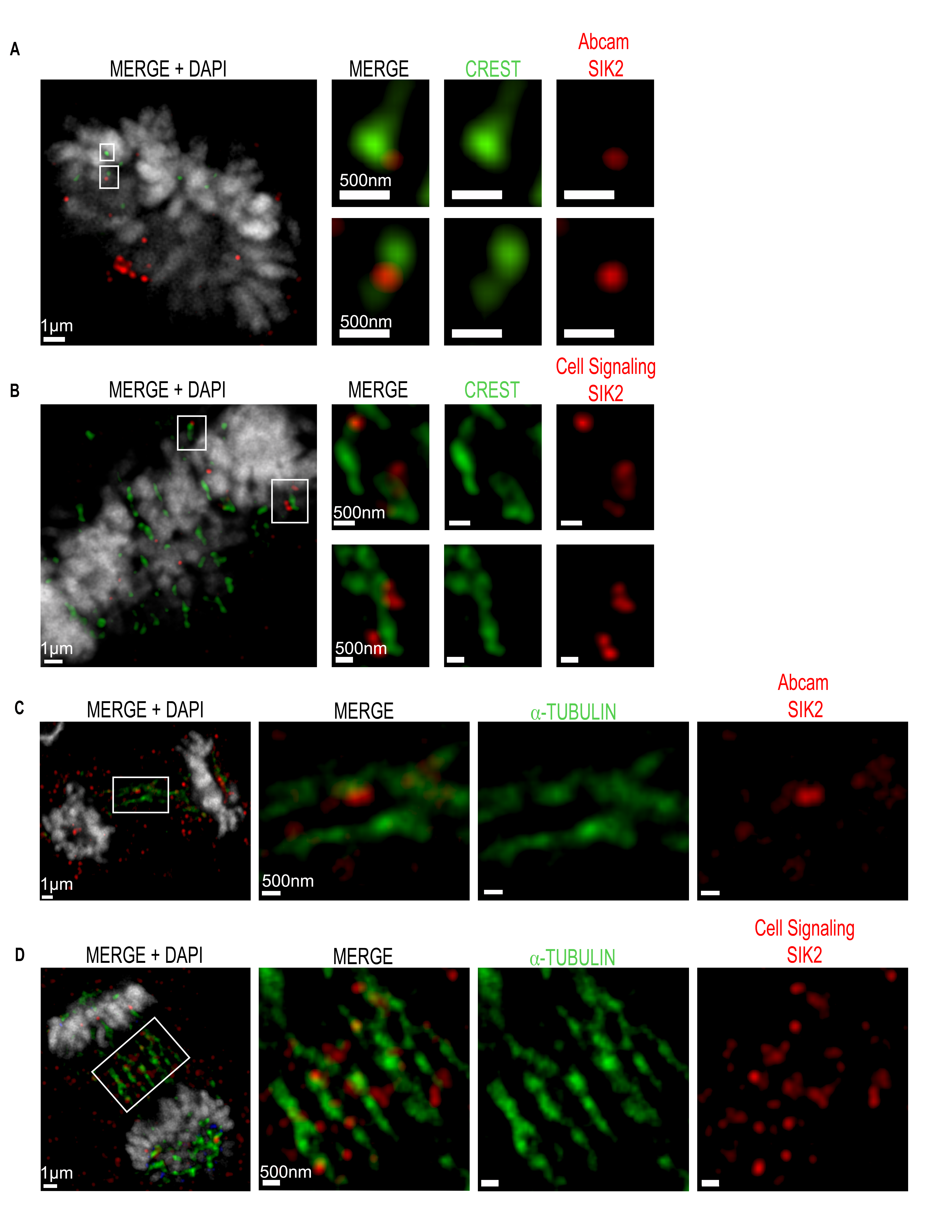

Supplement: Supplementary file 6 — Fig. S6. Validation of SIK2 localization. Representative immunofluorescence images show localization of SIK2 to mitotic centromeres (A, B) and central spindle (C, D) using with two unique antibodies (Abcam and Cell Signaling). Scale bars for whole cell images represent 1 μm. Scale bars for images of enlarged regions represent 500nm. [file MOL2-16-860-s007.tif]

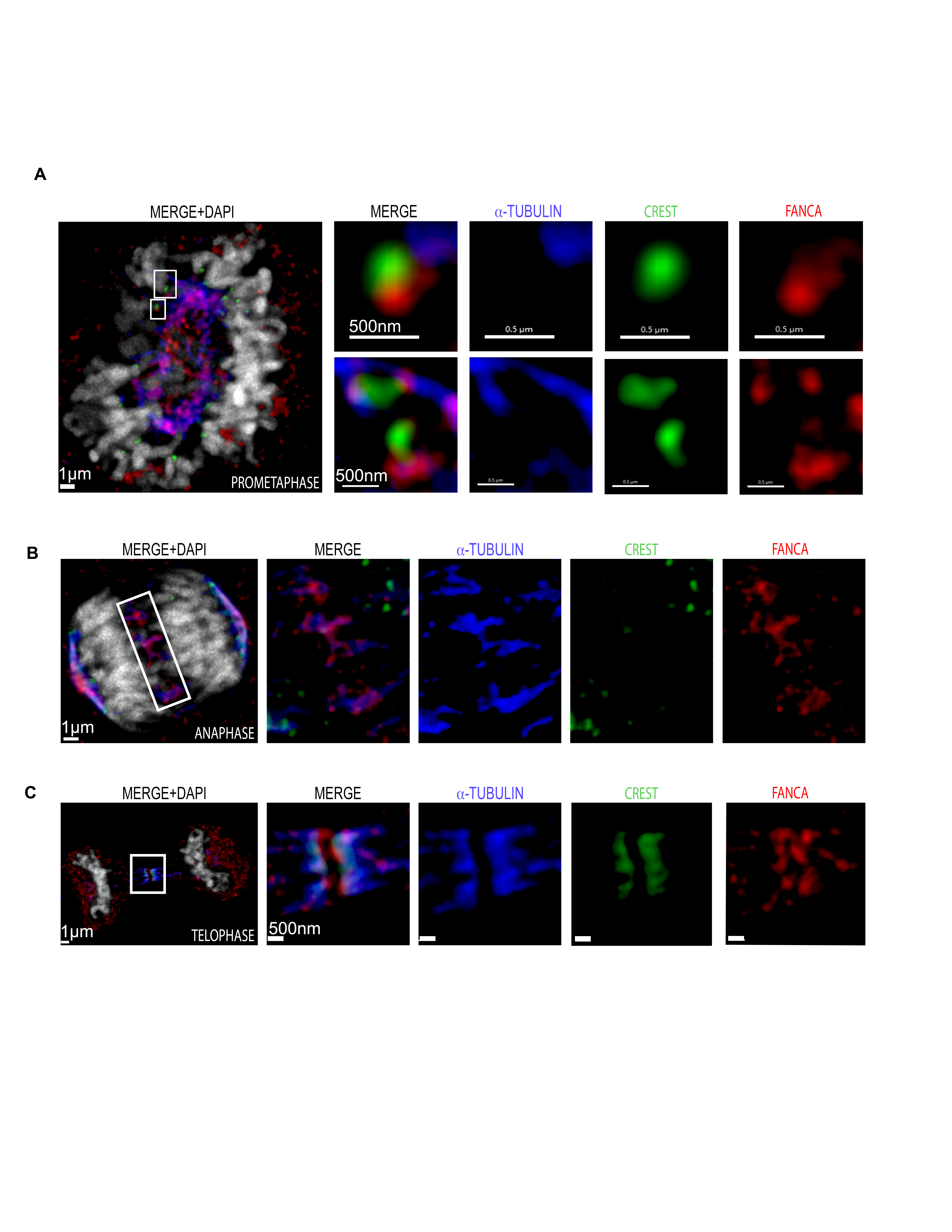

Supplement: Supplementary file 7 — Fig. S7. Validation of FANCA localization. (A–C) Representative immunofluorescence images show the localization of FANCA to mitotic centromeres, spindle, and central spindle using an additional FANCA antibody (Rabbit polyclonal; Abcam). Scale bars for whole cell images represent 1 μm. Scale bars for images of enlarged regions represent 500 nm. [file MOL2-16-860-s008.tif]
